# Multi‐Omics Reveals Dysregulated Neurotransmitter Systems in Aging and CNS Disorders

**DOI:** 10.1111/acel.70544

**Published:** 2026-05-13

**Authors:** Rui‐Ze Niu, Meng‐Yuan Zhang, Yan‐Ping Li, Xiao‐Qian Zhou, Xiao‐Lei Liu, Jie Wu, Wei‐Wei Wang, Yan‐Jun Wang, Yi Ruan, Yu Ding, Xiao‐Feng Zeng, Tian‐Hao Bao

**Affiliations:** ^1^ Affiliated Mental Health Centre of Kunming Medical University Kunming Yunnan China; ^2^ The Second People's Hospital of Honghe Prefecture Honghe Hospital of Yunnan Provincial Mental Hospital Honghe China; ^3^ Department of Neurology First Affiliated Hospital of Kunming Medical University Kunming China; ^4^ The Second Affiliated Hospital of Kunming Medical University Kunming Yunnan China; ^5^ School of Forensic Medicine Kunming Medical University Kunming Yunnan China

**Keywords:** aging, neuropsychiatric disorders, neurotransmitters, sex, single‐nucleus RNA sequencing

## Abstract

The neurotransmitter system (NTS) composed of neurotransmitter receptors and transporters is associated with a variety of diseases and disorders. The identification of disease‐associated NTS features is important for understanding disease mechanisms and for prioritizing potential therapeutic targets. The differences in the composition of NTS in aging and different diseases are not well understood. Here, we show how to integrate multi‐omics data at scale, including single‐cell and spatial transcriptomic, to identify disease‐associated NTS. By integrating, annotating, and analyzing 368 publicly available single‐nucleus RNA sequencing (snRNA‐seq) datasets from 17 cohort studies, we created a snRNA‐seq dataset for the prefrontal cortex (PFC) of the human brain. The resulting dataset included > 1 million cells covering healthy individuals throughout the life course after birth and eight common neuropsychiatric disorders. We comprehensively describe the cellular heterogeneity of NTS in aging and disease, and resolve the relationship between age or sex and disease‐associated NTS. A module composed of NTS and their regulatory genes was constructed and showed disease‐associated discriminatory signal, with supportive validation from protein‐level and external datasets. In order to screen NTS networks that can simulate disease brain tissue, we conducted a comparative analysis of five disease‐related cerebral organoids and found partial similarity between patient‐derived organoids and parental brain tissues. Our work provides a multi‐disease cell atlas based on large‐scale snRNA‐seq data and analyses the disease and sex heterogeneity of NTS, which provides new insights into the mechanisms of multiple CNS diseases and sheds light on disease precision therapy against NTS.

## Graphical abstract

Integrating large‐scale human snRNA‐seq, spatial transcriptomics, and cerebral organoid datasets reveals cell‐type‐specific neurotransmitter‐system remodeling across aging and CNS disorders. Shared and disease‐specific NTS alterations highlight candidate mechanisms, sex differences, and exploratory discriminatory signals that may guide mechanistic studies and therapeutic prioritization.

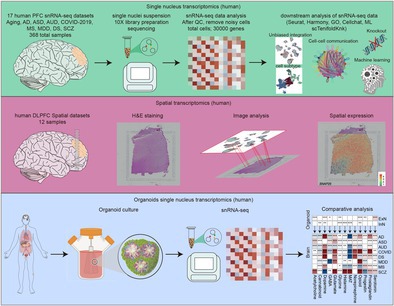

## 1 Introduction

Neurotransmitters (NT), such as dopamine, serotonin, GABA, and glutamate, are essential for transmitting signals across neurons and ensuring proper brain communication (Wu et al. 2022). The neurotransmitter system (NTS) refers to the network of neurons and their connections that use NT to communicate within the brain and the central nervous system (CNS). NTS plays a crucial role in maintaining normal brain function and its dysregulation is a key factor in the development of numerous neurological and psychiatric diseases (Chantranupong et al. 2023; Piot et al. 2023; Wang et al. 2023; Noviello et al. 2022). For example, in schizophrenia (SCZ), the dysregulation of neurotransmitters is a core pathological mechanism, mainly involving the dopamine, glutamate, GABA, and serotonin systems. Dopamine overactivity in SCZ is associated with positive symptoms (such as hallucinations and delusions), while insufficient dopamine function in the prefrontal cortex can lead to negative symptoms and cognitive impairment. GABA receptors undergo significant functional changes in various psychiatric disorders. In anxiety disorders, decreased GABA receptor function results in insufficient inhibitory neural conduction, leading to symptoms of hyperexcitability. The disruption of GABA receptors is associated with cognitive dysfunction in SCZ and depression. Positron emission tomography (PET) studies have found that the distribution of serotonin transporters (5‐HTT) has a greater impact on obsessive‐compulsive disorder, schizophrenia, and bipolar disorder compared to other receptors (Hansen et al. 2022).

Studies have shown that GluD1 receptors at inhibitory synapses can bind to GABA, resulting in a sustained enhancement of GABAergic synaptic currents through a non‐ionotropic mechanism (Piot et al. 2023). This indicates that the traditional dichotomy between glutamatergic and GABAergic receptors may no longer be applicable. Instead, examining the specific composition of NT in different cell types may provide a better understanding of their function and role in disease. Microglia are resident immune cells of the CNS that express a variety of neurotransmitter receptors (NTRs), including ionotropic glutamate receptors (iGluRs), metabotropic glutamate receptors (mGluRs), as well as GABA, dopamine, serotonin, and adrenergic receptors (Stowell and Wang 2025; Liu et al. 2016; Escoubas and Molofsky 2024). These receptors enable microglia to respond to neuronal signals and participate in maintaining brain homeostasis. For example, the activation of iGluRs, mGluRs, and purinergic receptors can influence microglial activation, proliferation, and the release of pro‐inflammatory cytokines (Bi et al. 2026). In contrast, the activation of GABA, dopamine D2, and serotonin receptors generally reduces the pro‐inflammatory activity of microglia and promotes neuroprotective effects (Bi et al. 2026; Lee 2013). Emerging evidence suggests that microglia and the serotonergic system form a bidirectional regulatory axis in the CNS. Pro‐inflammatory microglial activation can impair serotonergic neurotransmission, while serotonin receptor‐dependent signaling modulates microglial phenotype and inflammatory responses, together contributing to the pathogenesis of neuropsychiatric and neurodegenerative disorders (Zheng and Xu 2025). Furthermore, astrocytes also express a variety of NTRs, including glutamate, GABA, and dopamine receptors (Kofuji and Araque 2021). Through these receptors and related transporters, astrocytes take up excess neurotransmitters from the synaptic cleft and help regulate synaptic and neuronal electrical activity (Haydon and Carmignoto 2006; Allen 2014; Choi et al. 2026). Additionally, astrocytes can respond to neurotransmitters by releasing gliotransmitters or undergoing phenotypic changes, thereby modulating microglial activation and neuroinflammatory responses (Covelo and Araque 2018; Faust et al. 2025). Recent evidence further shows that dopamine can directly regulate astrocyte‐mediated synapse remodeling through MEGF10‐dependent phagocytic pathways, highlighting an active role for astrocytes in neurotransmitter‐driven circuit plasticity (Choi et al. 2026). These results suggest that focusing on cell‐type‐specific differences in NT composition and cell‐to‐cell regulatory differences could enhance our understanding of the development of psychiatric disorders.

Aging is accompanied by progressive changes in neurotransmitter receptors and transporters across multiple brain cell types, providing an important biological context for interpreting disease‐associated NTS alterations. In this study, we therefore used aging as a comparative framework rather than assuming that all included disorders are equally aging‐related. Importantly, different CNS disorders also show distinct age‐specific patterns of onset and progression. For instance, AD occurs predominantly in older adults, whereas SCZ is more common from adolescence to mid‐adulthood, ASD emerges mainly in childhood and adolescence, and MDD frequently begins in early or mid‐adulthood. Moreover, some disorders not classically categorized as aging‐related may still intersect with brain aging biology; for example, DS and SCZ have both been linked to accelerated brain aging (Niu et al. 2025; Constantinides et al. 2023; Fu et al. 2025), while COVID‐19 has raised concern because of its effects on brain function and its potential association with aging‐like brain changes (Corrigan et al. 2024; González‐Rosa et al. 2024). Under this framework, we sought to determine which NTS alterations are shared across disorders and which are specific to particular pathological settings. NTS have been implicated in a variety of diseases and disorders. Identifying specific NTS associated with particular diseases is important for understanding disease mechanisms and for guiding the development of targeted therapeutic strategies. Thus, we aimed to investigate how changes in various NTRs and transporters across different cell types are linked to a range of CNS disorders across aging and disease contexts. By integrating large‐scale human prefrontal single‐cell transcriptome data, we evaluated the expression changes of 13 common neurotransmitter receptors and transporters across seven diseases and the aging process. Throughout life, each neurotransmitter receptor (NTR) exhibits distinct trends and magnitudes of fluctuation across various cell types. In different diseases, the expression of a single NTR may follow similar patterns, reflecting a shared biochemical basis for common symptoms across diseases. For instance, AD and DS cluster together, consistent with the clinical observation of a high risk of developing AD in individuals with DS. Similarly, COVID‐19 and AD cluster together, suggesting a potential future risk of AD in COVID‐19 patients. The constitutive expression of various NTRs across different cell types varies between diseases, providing a framework for identifying disease‐associated features and potential therapeutic targets. In conclusion, given the variability of NTS across psychiatric disorders, assessing their dynamics in both health and disease using in vivo tools with high sensitivity and spatiotemporal resolution may improve our understanding of disease progression and support future translational studies.

## 2 Results

### 2.1 Large‐Scale Unbiased Integration of Human PFC snRNA‐Seq Dataset Across Aging and Eight Common CNS Disorders

**FIGURE 1:**
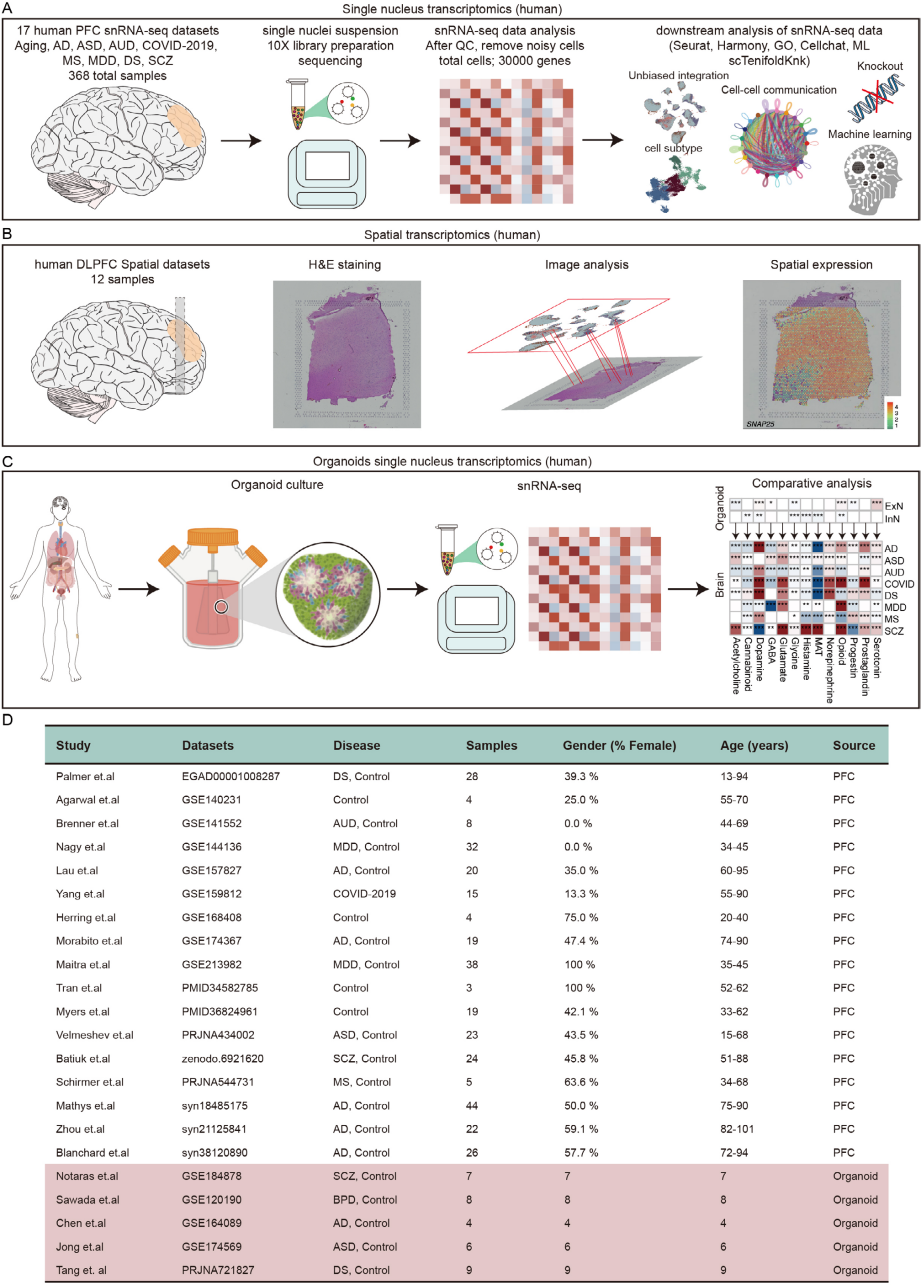
Overview of snRNA‐seq and spatial transcriptomics analysis workflows for the human brain PFC. (A) Workflow of data generation, integration, and annotation for the human brains' snRNA‐seq datasets. (B) Spatial transcriptomics profiling workflow of human brains. (C) snRNA‐seq analysis workflows for the human cerebral organoids. *FDR*‐corrected *p* values are indicated by asterisks, ***p < 0.001, **p < 0.01, *p < 0.05. (D) Metadata table for all datasets included in this study. AD, Alzheimer's disease; ASD, autism spectrum disorder; AUD, alcohol use disorder; COVID‐19, corona virus disease 2019; DLPFC, dorsolateral prefrontal cortex; DS, Down syndrome; MDD, major depressive disorder, MS, multiple sclerosis; PFC, prefrontal cortex; QC, quality control; SCZ, schizophrenia; snRNA‐seq, single‐nucleus RNA‐sequencing.

**FIGURE 2:**
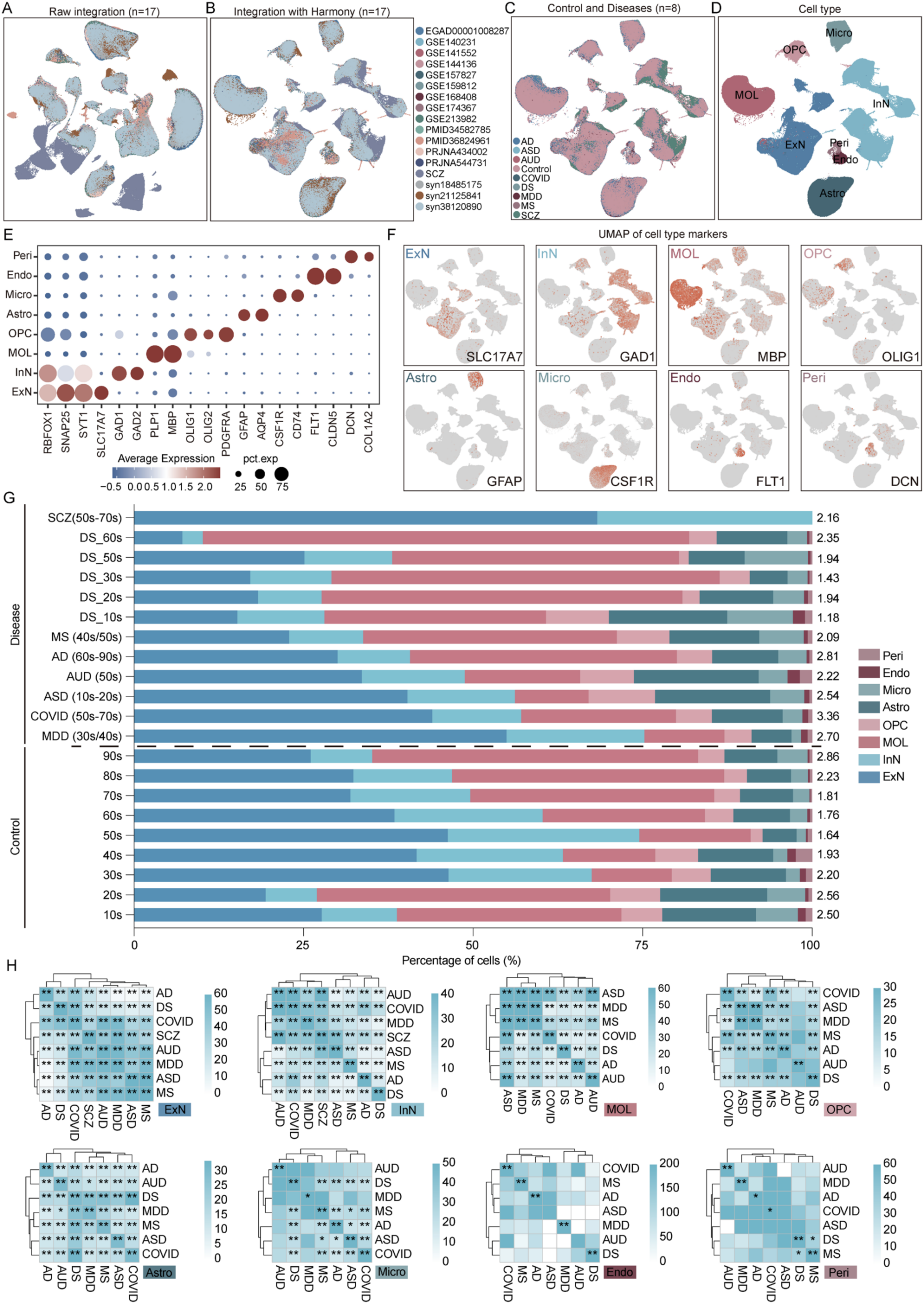
Integration, cell‐type identification, and distribution of snRNA‐seq data across multiple datasets and conditions. (A) Raw integration of 17 datasets without batch correction showing batch effects across datasets. (B) Batch‐corrected integration using Harmony displaying effective alignment of datasets while preserving biological variation. (C) UMAP of cells grouped by disease and control conditions (*n* = 8 diseases), showing clustering across AD, ASD, DS, SCZ, COVID‐19, MS, AUD, MDD, and healthy controls. (D) Cell‐type annotation of integrated snRNA‐seq data based on marker genes, identifying major cell types: ExN (excitatory neurons), InN (inhibitory neurons), Astro (astrocytes), Micro (microglia), OPC (oligodendrocyte precursor cells), MOL (mature oligodendrocytes), Endo (endothelial cells), Peri (pericytes). (E) Dot plot of marker gene expression for each annotated cell type, showing average expression and percentage of cells expressing each marker gene. (F) UMAP visualization of key cell type‐specific marker genes. (G) Bar plot of cell‐type proportions across disease and control samples, stratified by age. The number on the right represents the ratio of excitatory and inhibitory neurons. (H) Heatmaps showing the overlap (OR, one‐sided Fisher's exact test) of genes significantly alterative associated with pathology between the diseases indicated for ExN, InN, Astro, Micro, OPC, MOL, Endo, and Peri. **log_10_(*p* value) > 200, *log_10_(*p* value) > 100 (one‐sided Fisher's exact test).

Research strategy includes collecting human brain snRNA‐seq, cerebral organoids snRNA‐seq, spatial transcriptomic, and proteomic datasets to avoid the limitations of single methodology (Figure 1A–D). Briefly, we performed quality control and integration for PFC snRNA‐seq datasets from healthy individuals and eight CNS diseases (AD, ASD, AUD, COVID‐19, DS, MDD, MS, and SCZ) brain tissues (Figure 1A, Table S1). To provide a high‐resolution reference map of regional preferences, we included the spatial transcriptome dataset of human dorsolateral prefrontal cortex (DLPFC) (Figure 1B) (Maynard et al. 2021). To assess the efficacy of patient‐derived cerebral organoids in mimicking parental brain tissues, we included snRNA‐seq data from six datasets for five diseases (AD, ASD, BPD, SCZ, and DS) (Figure 1C) (Chen et al. 2021; de Jong et al. 2021; Sawada et al. 2020; Notaras et al. 2022; Tang et al. 2021). In total, we integrated 368 publicly available human PFC snRNA‐seq datasets from 17 cohort studies (Figure 1D). After removing cells of low quality and doublets, the resulting dataset consisted of > 1 million nuclei (Figure 2A). Due to differences in specimen sources, batch effects were evident among these data (Figure 2A). After integration, the batch effects were satisfactorily removed (Figure 2B,C and Figure S1). Although all datasets included in the main analysis were derived from the prefrontal cortex, we acknowledge that residual heterogeneity among PFC subregions across studies may still contribute to variability; therefore, batch correction and quantitative integration benchmarking were performed to minimize study‐specific effects. For integrated datasets, nuclei were manually categorized into eight major cell types using canonical marker genes including excitatory (ExN) and inhibitory (InN) neurons, astrocytes (Astro), microglia (Micro), mature oligodendrocytes (MOL), oligodendrocyte progenitor cells (OPCs), endothelial cells (Endo), and pericytes (Peri) (Figure 2D–F). With the exception of SCZ, which was sorted by NeuN, the other datasets included all cell types (Table S2 and Figure S1B). Therefore, in downstream analysis, SCZ only focused on ExN and InN, while all cell types were analyzed for other diseases. The snRNA‐seq datasets analyzed here can be interrogated with an interactive web interface (http://brainpfcatlas.cn/).

### 2.2 Cellular Composition and Molecular Differences Across Brain Aging and Eight Common CNS Disorders

According to the age at the time of sample acquisition, we divided all samples into different age groups with a span of 10 years (10s–90s) (Figure 2G). The cell composition showed a trend change during aging, such as the decrease of neurons and the increase of glial cells (Figure 2G). The DS, however, has no such change. There were also differences in cell composition among different diseases compared with healthy controls (Figure 2G). Because the number of cells, especially Micro and MOL, may be affected by sample handling and sequencing methods, we mainly focused on the ratio differences between ExN and InN (Figure 2G). In healthy individuals, the ratio of ExN and InN peaked in the 20s, followed by a decline into the 50s, after which they experienced a subsequent increase (Figure 2G). Each age group in DS was lower than the corresponding control group, and the decline was earlier (Figure 2G). DS in the early stage (10s) was significantly lower than that in the control group, suggesting abnormal development in the early stage of DS. Compared with the control group, although the proportion of ExN and InN did not change significantly in ASD, the proportion of neurons and glial cells was significantly abnormal. In SCZ, MS, AD, AUD, and MDD, the proportion of ExN and InN was significantly abnormal, showing premature aging (Figure 2G). The proportion of ExN and InN increased significantly in COVID‐19, suggesting the effect of viral invasion on specific neurons (Figure 2G). In addition, we performed subtype analyses and comparisons for ExN, InN, Astro, Micro, and Endo (Figure S1). We found that the *CD44* ^+^ reactive Astro and *CTSB* ^+^ *SPP1* ^+^ activated Micro were increased in AD, DS, MS, and COVID (Figure S2C,D).

To assess transcriptomic alterations in different cell types across diseases, we used model‐based analysis of single‐cell transcriptomics (MAST) to analyze differentially expressed genes (DEGs) between disease and age‐matched healthy controls (Figure 2H). When comparing DEGs associated with different disease types, we observed significant overlap in disease‐associated DEGs in ExN, InN, MOL, and Astro (Figure 2H). There was no significant overlap between the DEGs of Endo and Peri in different diseases. Consistent with previous studies, cluster analysis based on DEGs shows that AD and DS cluster together (Figure 2H).

### 2.3 Changes of NTS in Different Cell Types Across the Postnatal Lifespan

**FIGURE 3:**
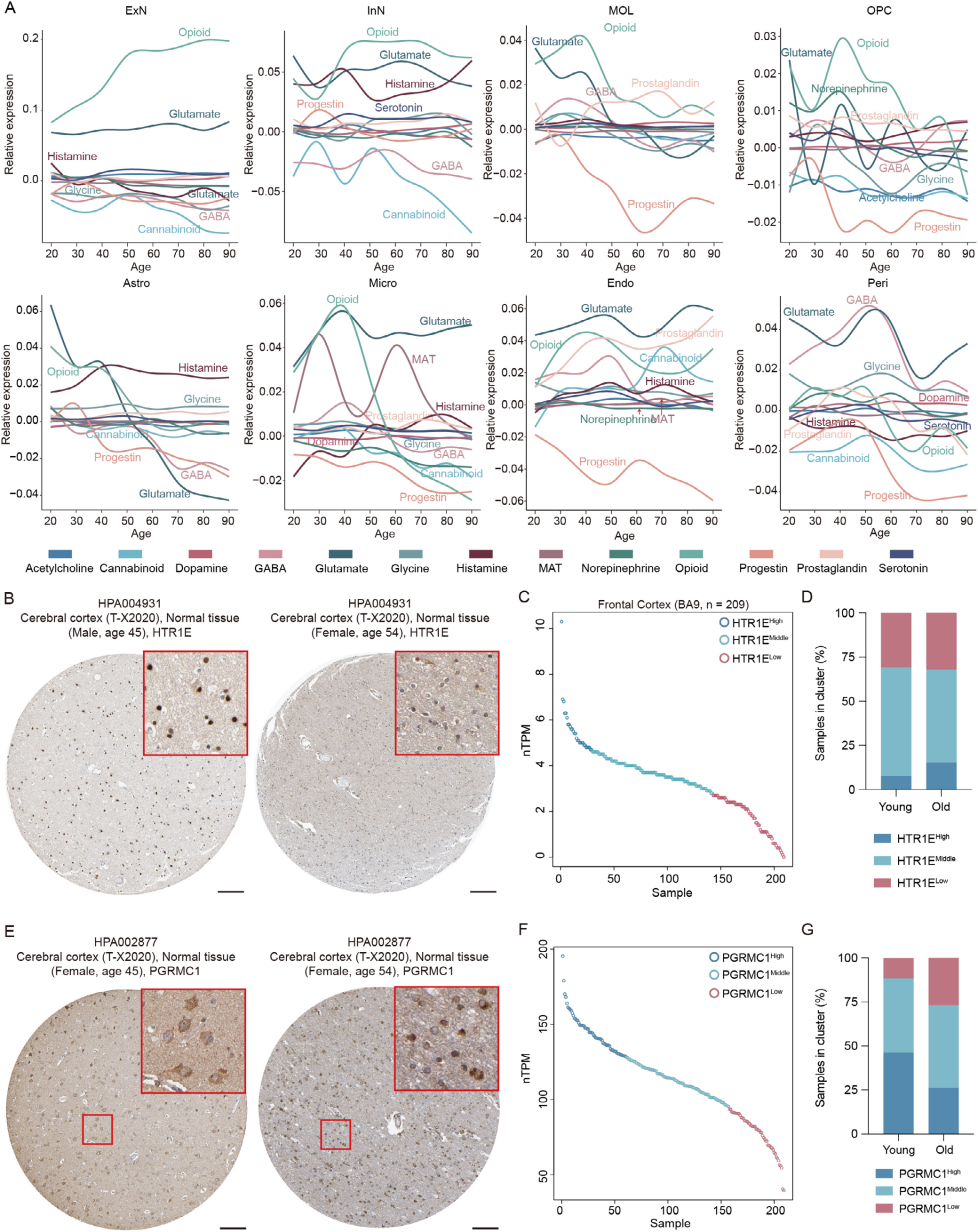
Age‐dependent NTS molecule expression patterns across cell types. (A) Relative expression of NTS across cell types (ExN, InN, MOL, OPC, Astro, Micro, Endo, Peri) as a function of age. NTS include acetylcholine, cannabinoid, dopamine, GABA, glutamate, glycine, histamine, MAT (monoamine transporters), norepinephrine, opioid, progestin, prostaglandin, and serotonin. Notable trends include age‐associated increases or decreases in expression levels of specific neurotransmitters within distinct cell types. (B) Immunohistochemical staining of HTR1E in cerebral cortex tissue (HPA004931). Images show representative staining in male (age 45) and female (age 54) samples. Scale bar: 100 μm. (C) Scatter plot of normalized TPM (nTPM) values showing HTR1E expression levels in frontal cortex samples (BA9, *n* = 209). Samples are stratified into three clusters: HTR1E^high^, HTR1E^middle^, and HTR1E^low^. (D) Bar plot showing the distribution of HTR1E expression clusters across young and old groups. The proportion of samples in each cluster is displayed. (E) Immunohistochemical staining of PGRMC1 in cerebral cortex tissue (HPA002877). Images show representative staining in female (age 45) and female (age 54) samples. Insets highlight specific regions with detailed staining patterns. Scale bar: 100 μm. (F) Scatter plot of normalized TPM (nTPM) values showing PGRMC1 expression levels in frontal cortex samples (BA9, *n* = 209). Samples are stratified into three clusters: PGRMC1^high^, PGRMC1^middle^, and PGRMC1^low^. (G) Bar plot showing the distribution of PGRMC1 expression clusters across young and old groups. The proportion of samples in each cluster is displayed.

In order to evaluate the changes of different NTR‐related genes across lifespan, we collected the receptor and transporter genes of 13 NTS (Table S3). In total, we analyzed 173 genes derived from acetylcholine (27 genes), cannabinoid (3), dopamine (7), gamma‐aminobutyric acid (GABA) (30), glutamate (42), glycine (6), histamine (4), monoamine transporter (MAT, 2), norepinephrine (10), opioid (5), progestin (9), prostaglandin (10), and serotonin (18). With 10s as reference, we analyzed the changes of 13 NTS in each age group (Figure 3A). The expression abundance of different NTS was different in cell types. Opioid was the most abundant in neurons, MOL and OPC (Figure 3A), while glutamate was the most abundant in Micro and Endo (Figure 3A). Glutamate and opioid showed consistent changes throughout the entire life process. Histamine has a high expression level in InN and Astro (Figure 3A). Progestin decreased significantly with age in all cell types. Prostaglandin was highly expressed in Endo and increased with age. Studies have shown that prostaglandin E2 (*PGE2*) is a major regulator of inflammation (Minhas et al. 2021; Yin et al. 2022; Serrats et al. 2017). The changes of NTS in Endo and Peri reflect its role in regulating the balance between peripheral and central (Figure 3A).

Then, in each cell type, we performed cluster analysis and timing analysis for these 173 genes (Figures S3 and S4). Most of the genes showed age‐dependent increases or decreases. From the trend of transcriptomic changes, we could find that around age 50 may represent a key transition stage of NTS change (Figures S3 and S4). Immunohistochemical staining images and frontal cortex RNA‐seq data from the Human Protein Atlas (HPA) provided supportive cross‐dataset validation for the snRNA‐seq findings. In particular, age‐associated increases in *HTR1E* and decreases in *PGRMC1* observed in snRNA‐seq were further supported by representative HPA immunohistochemical images and by expression stratification analysis of 209 frontal cortex (BA9) samples from HPA (Figure 3B–G). The expression of *HTR1E* in ExN and InN increased after age 50 (Figure S3). *HTR1E* positive cells were significantly increased in cortical neurons (Figure 3B). A cluster analysis of RNA‐seq data from 209 samples was performed. According to the expression of *HTR1E*, we divided 209 samples into three expression groups (Figure 3C). Further dividing the 209 samples into young (< 50) and old groups (> 50) according to age, we found that the proportion of *HTR1E* ^high^ group increased significantly in the old group (Figure 3D). *PGRMC1* positive cells were significantly decreased in cortical neurons (Figure 3E). A cluster analysis showed that the proportion of *PGRMC1* ^high^ group decreased significantly in the old group, while *PGRMC1* ^low^ group increased significantly in the old group (Figure 3F,G).

### 2.4 Changes of NTS in Different Cell Types Across Disease

**FIGURE 4:**
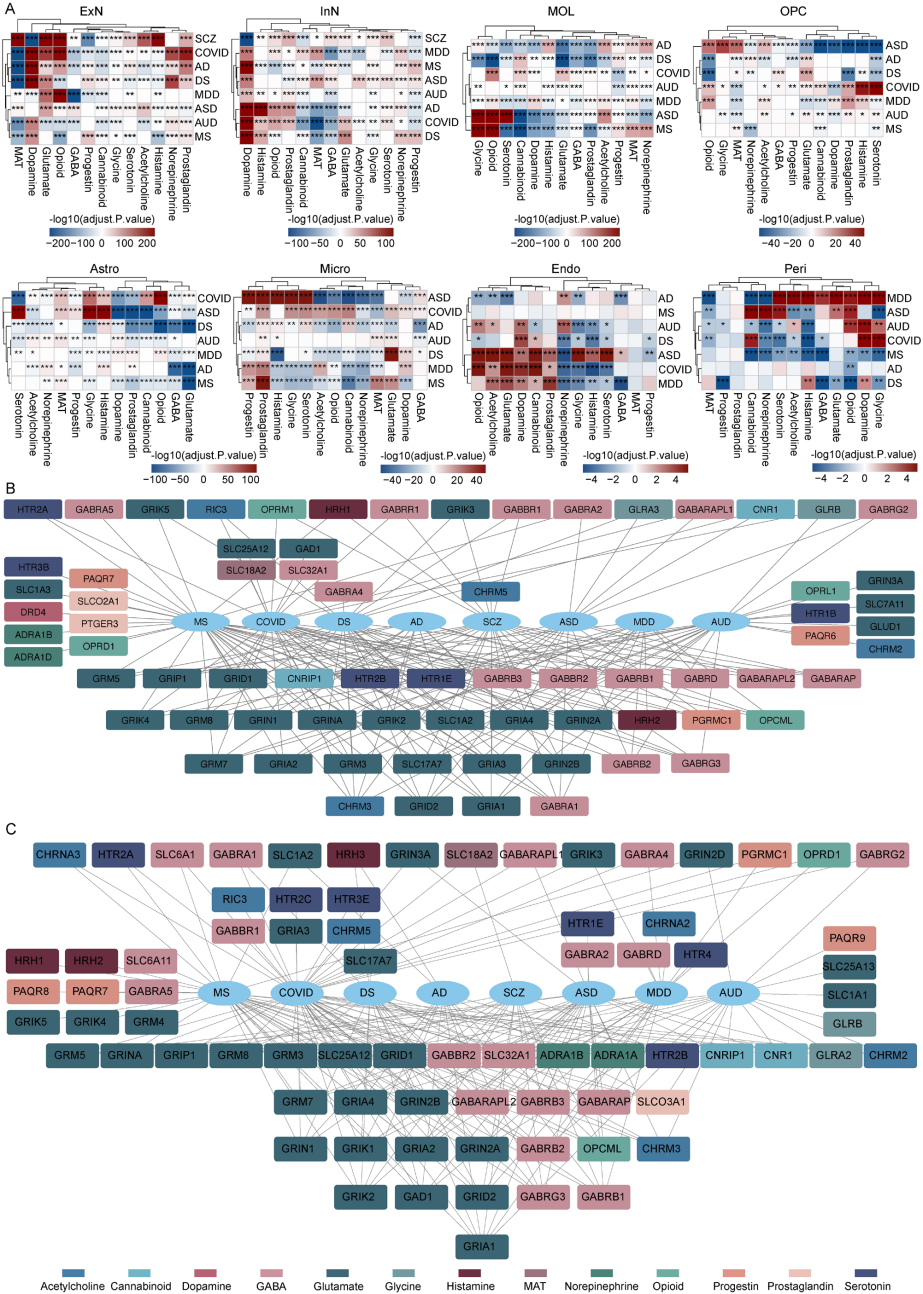
Disease‐specific alterations in NTS and cross‐disease signaling networks. (A) Heatmaps showing the enrichment score differences of various NTS in different diseases (SCZ, COVID, AD, ASD, MDD, MS, AUD, DS) for different cell types (ExN, InN, MOL, OPC, Astro, Micro, Endo, Peri). Color indicates the log_10_‐transformed adjusted *p* values of enrichment scores, with red indicating upregulation and blue indicating downregulation. Asterisks denote the significance levels after FDR correction. (B) Network visualization of NTS‐related genes that are differentially expressed in ExN across different diseases. (C) Network visualization of NTS‐related genes that are differentially expressed in InN across different diseases. Nodes represent genes, color‐coded by neurotransmitter systems, and edges indicate gene–gene interactions or co‐expression relationships.

NTS have been implicated in a variety of diseases and disorders. Therefore, we assessed 13 NTS variations in eight common CNS diseases. Geneset score and cluster analysis showed that AD and DS cluster together (Figure 4A). COVID‐19 tends to be in the same category as AD and DS (Figure 4A). SCZ occurs in a single branch and is significantly different from other diseases (Figure 4A). Research suggests that the negative/deficit symptom complex in schizophrenia may be related to low dopamine activity in the prefrontal cortex (Davis et al. 1991). We found that dopamine NTS was significantly reduced in ExN and InN (Figure 4A). Most NTS are elevated in SCZ (Figure 4A). The Cellchat analysis yielded the same results (Figure S5). The reduced specificity of dopamine NTS in SCZ neurons can be used as a basis for differential diagnosis of SCZ compared to other diseases. Studies have shown that increased *PGE2* signaling inhibits the bioenergy of microglia, leading to an unadapted pro‐inflammatory response (Minhas et al. 2021). We found that the proportion of activate‐related microglia (ARM) increased in DS, COVID‐19, MS, and AD, consistent with an increase in prostaglandin NTS (Figure 4A). The differential change of NTS in different cells alters the communication between cells. Therefore, we analyzed cellular communication between cell subtypes of ExN and InN. The overall number of cell communications was significantly reduced in all diseases compared to age‐ and sex‐matched controls. In addition to the increased communication strength of ASD, COVID‐19, and SCZ, the communication strength of other diseases decreased (Figure S6). The number and intensity of cellular communication between cell subpopulations varies across diseases.

**FIGURE 5:**
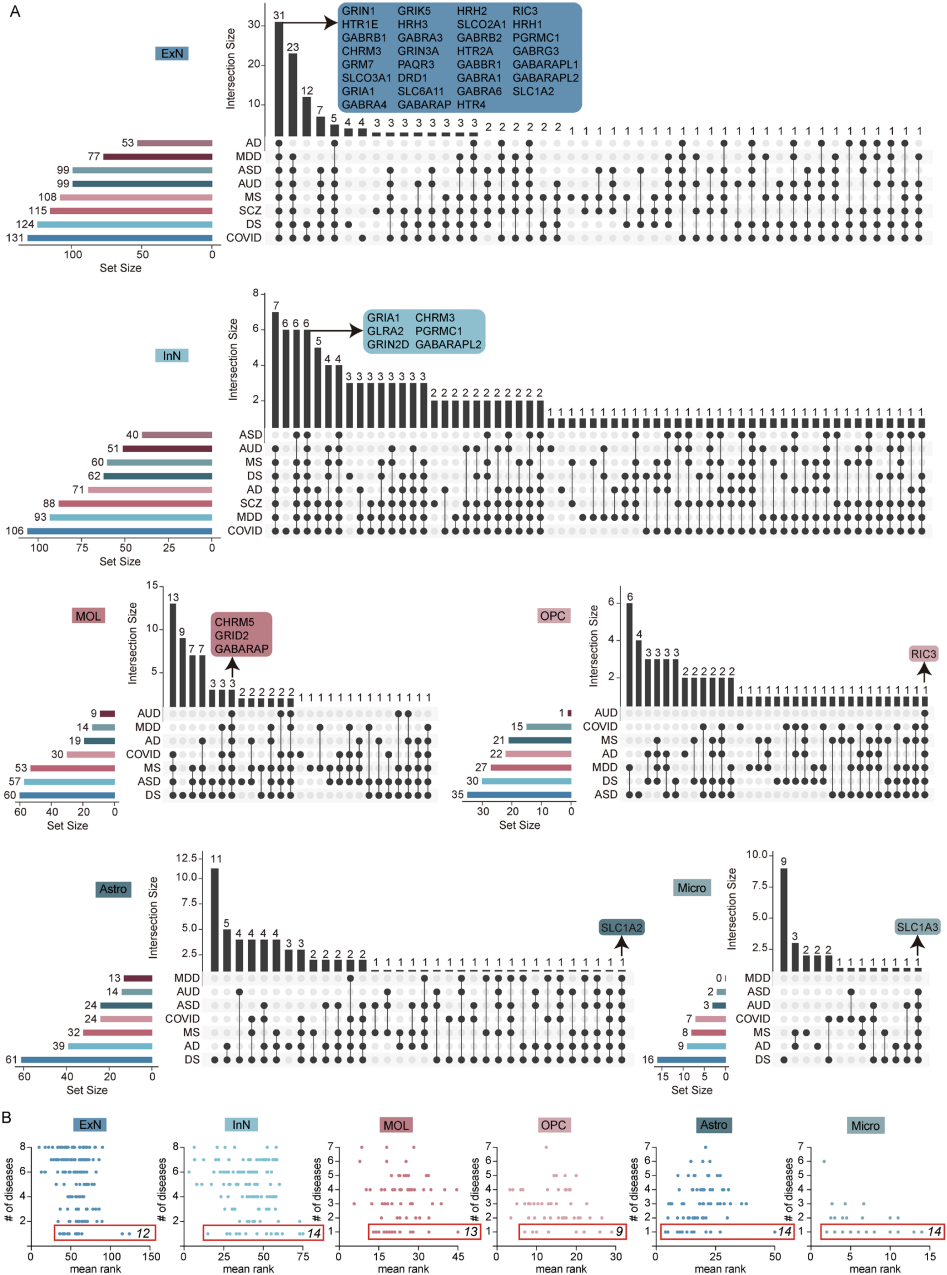
Shared and cell type‐specific differentially expressed NTS‐associated genes across disorders. (A) UpSet plots showing intersections of NTS‐related genes across diseases within each cell type (ExN, InN, MOL, OPC, Astro, Micro). Top shared genes are highlighted in each cell type. (B) Scatter plots summarizing the number of diseases and mean rank of shared genes across diseases for each cell type. Disease‐specific genes are highlighted.

We then analyzed NTS‐associated genes in disease‐associated DEGs (Table S4). Our results indicate that most genes are shared among the diseases, with only a few being disease‐specific (Figures 4B,C and 5, and Figure S6). MS, AUD, and COVID‐19 have more specific NTS‐related DEGs (Figure 4B and Figure S6), which reflects the mechanism specificity of organic diseases and psychoactive substance‐induced mental disorders. The age‐specific expression abundance and variation of NTRs form the basis of disease occurrence. For example, AD‐related differential NTRs (91.6%) expressed higher abundance during the 50s–90s, and ASD‐related differential NTRs (50%) expressed higher abundance before 30s (Figure 4B and Figure S3A). Studies have shown that at the population level, patients with SCZ and MDD show signs of premature aging (Constantinides et al. 2023; Han et al. 2021). Indeed, SCZ and MDD‐related differences NTRs express higher abundances (44.4% and 71.4%) after 50s (Figure 4B and Figure S3A). In addition, we found the highest number of differentially expressed NTS genes in COVID‐19 neurons, reflecting the severity of viral infection. Most of the NTS genes are differentially altered in at least two diseases, and only a few genes are differentially expressed in a single disease (Figure 5B).

### 2.5 The Spatial Hierarchical Distribution Map of NTS Within the PFC

**FIGURE 6:**
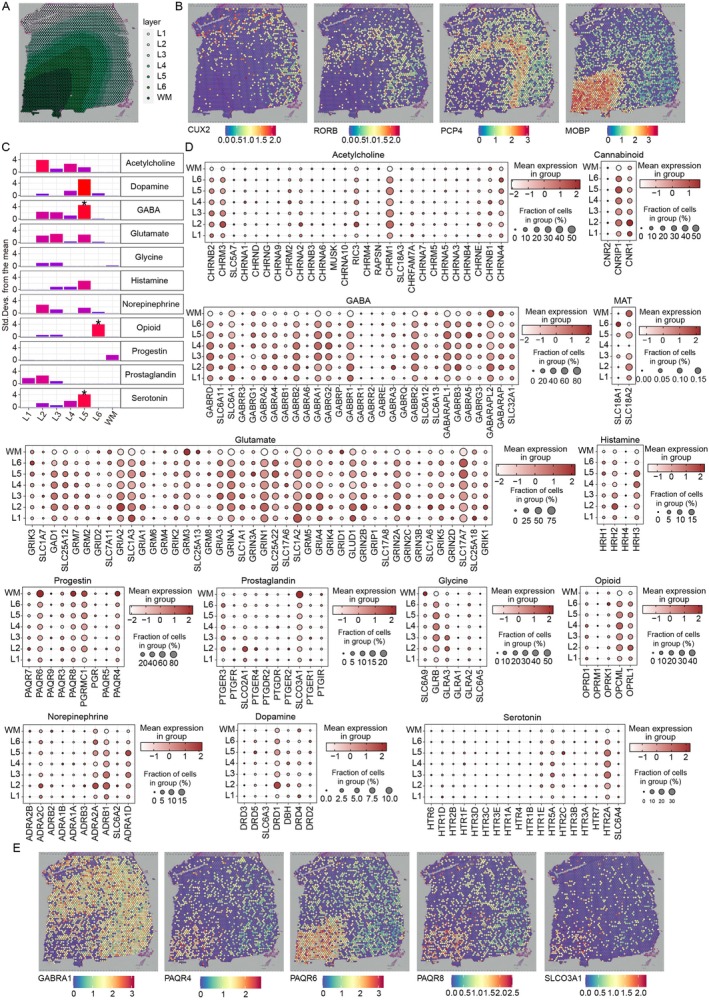
The spatial distribution map of NTS in PFC. (A) The hierarchical classification of PFC samples within spatial transcriptome data encompasses layers L1 (Layer 1) through L6 (Layer 6), as well as white matter (WM). Distinct colors denote various hierarchical regions, thereby providing a spatial reference. (B) Spatial expression patterns of cortical marker genes, including CUX2 (L2), RORB (L4), PCP4 (L5), and MOBP (WM), are illustrated. Different colors represent the intensity of gene expression; red indicates high expression levels while blue signifies low expression levels. (C) Preferences for cortical distribution among NTS‐related genes are examined. (D) A dot plot depicting the expression of NTS‐related genes is presented; the size of each dot corresponds to the proportion of expressing cells within the group (fraction of cells in group), while the color reflects the average gene expression level in that group (mean expression in group). (E) The spatial distribution of GABRA1, PAQR4, PAQR6, PAQR8, and SLC30A1 expressions is shown; color coding indicates varying levels of gene expression with red representing high levels and blue indicating low levels.

To analyze the spatial distribution preferences of NTS, we conducted a detailed examination of the spatial transcriptome data from 12 prefrontal cortices (PFCs) (Figure 6). Utilizing cortical marker genes, we were able to distinctly categorize the PFC into six cortical layers (L1‐L6) and white matter (WM) regions (Figure 6A). CUX2 is predominantly expressed in superficial neurons located in layers 1–3, while RORB is primarily found in intermediate layer neurons. Additionally, PCP4 and MOBP are specifically associated with deep neurons and myelin, respectively (Figure 6B). Subsequently, we assessed the distribution preferences of various NTS within the cortex (Figure 6C,D). Notably, excitatory neurotransmitters such as glutamate and inhibitory neurotransmitters like GABA exhibit high expression levels in intermediate layer neurons, suggesting that these layers may serve as critical regions for information integration (Figure 6D,E). The distribution patterns of specific metabolism‐related genes alongside myelin‐related genes further underscore the significance of white matter regions in neural support and myelin maintenance. Progestin shows predominant expression within WM areas (Figure 6D,E), indicating its potential role in diseases characterized by myelin abnormalities such as AD, MS, and AUD, which have been reflected through disease‐associated NTS findings presented earlier (Figure 4B,C).

### 2.6 The Potential Ability of Cerebral Organoids to Mimic Disease

**FIGURE 7:**
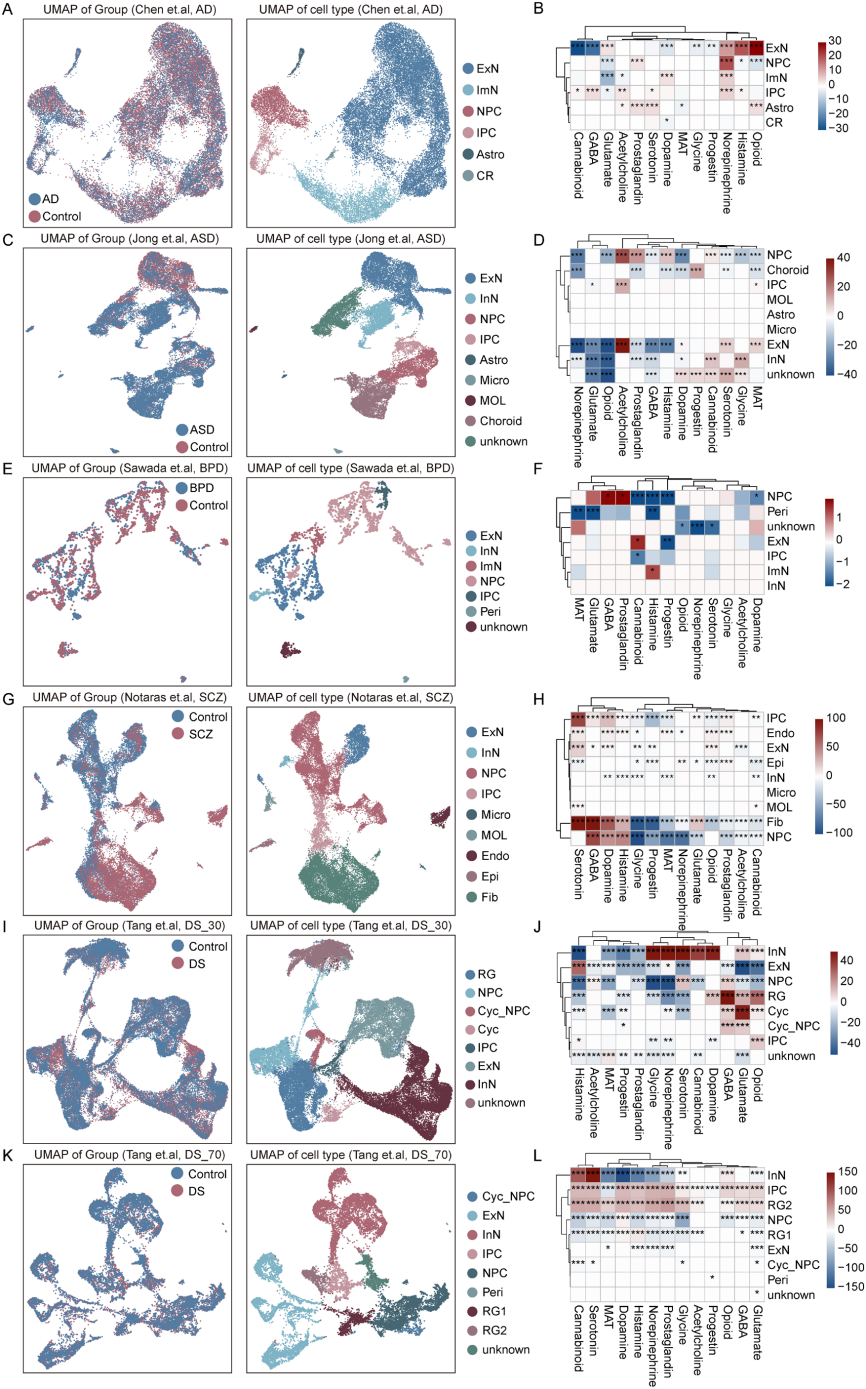
UMAP visualization and NTS differences in organoid‐derived samples for specific diseases. (A–L) UMAP projections and heatmaps for groups and cell types in AD, ASD, BPD, SCZ, and DS datasets. (A, C, E, G, I, K) UMAPs display clustering of control versus disease groups and cell types. (B, D, F, H, J, L) Heatmaps illustrate disease‐specific changes in NTS across cell types, *FDR*‐corrected *p* values are indicated by asterisks, ***p < 0.001, **p < 0.01, *p < 0.05.

To evaluate the efficacy of disease‐associated organoids in mimicking the parental diseases, we collected six snRNA‐seq datasets from five conditions, including AD, ASD, BPD, SCZ, and DS (Figure 7). Because the psychiatric datasets analyzed here were derived from independent cerebral organoid studies, differences in donor background, induction protocol, and maturation stage may contribute to variation in cell‐type composition and transcriptional patterns across datasets, although major shared populations such as ExN, InN, IPC, and NPC were consistently identified using the same validated marker genes. The difference of NTS in SCZ and DS is more complex than in other diseases, reflecting more significant early developmental abnormalities in SCZ and DS (Figure 7). We focused on the similarity of ExN or InN in organoids and brain tissue. We found consistent changes of NTS in AD organoids and brain tissue (Figure 7A,B). Glutamate, histamine, and opioid were increased in organoids and brain tissue of AD, while cannabinoid, GABA, and glycine were decreased. Acetylcholine, prostaglandin, histamine, serotonin, and MAT showed consistent and significant changes in the organoids and brain tissues of ASD (Figure 7C,D). The depressive phase of bipolar disorder (BPD) shows symptoms of depression such as slow thinking, reduced language, and negative emotions. We found that cannabinoid and progestin of ExN change uniformly in the organoids of BPD and the brain tissue of MDD (Figure 7E,F). In SCZ ExN, serotonin, GABA, and opioid showed consistent expression in organoids and brain tissue (Figure 7G,H). In InN, dopamine and cannabinoid are consistent in organoids and brain tissue of SCZ (Figure 7G,H). In DS ExN, acetylcholine, MAT, norepinephrine, serotonin, and opioid showed consistent changes in organoids at 30 days and brain tissue of DS (Figure 7I,J). In InN, MAT, norepinephrine, dopamine, and opioid changed uniformly in organoids at 30 days and brain tissue of DS (Figure 7I,J). However, NTS changed significantly in organoids at day 70 compared to day 30 (Figure 7K,L).

### 2.7 Sex Differences of NTS in Different Diseases

**FIGURE 8:**
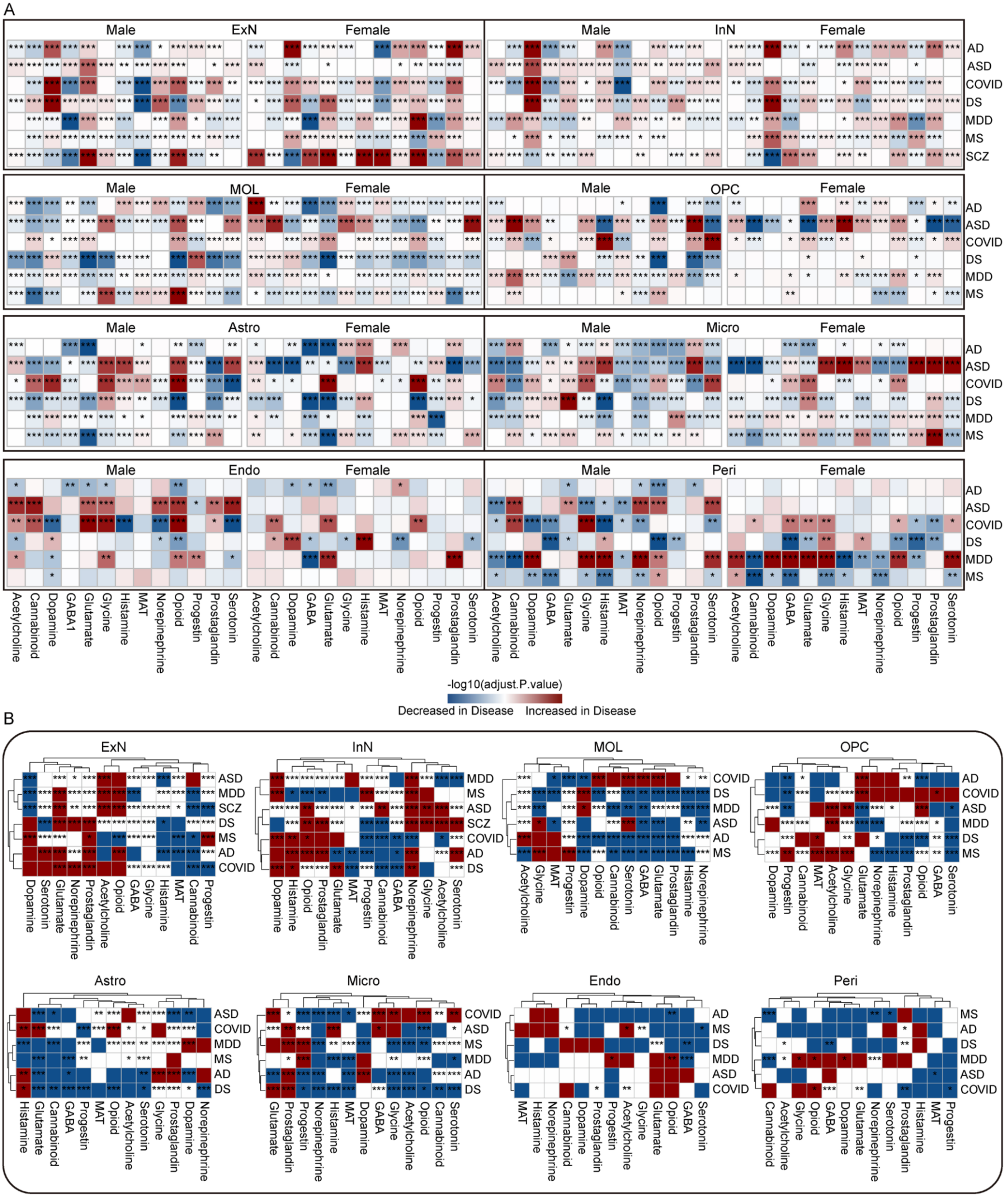
Sex‐specific alterations for NTS across diseases. (A) Heatmaps of NTS differences across male and female samples within each disease group (AD, ASD, COVID, DS, MDD, MS, SCZ). Differences are shown for ExN, InN, MOL, OPC, Astro, Micro, Endo, and Peri. (B) Comparison of NTS alterations across diseases, highlighting significant differences. *FDR*‐corrected *p* values are indicated by asterisks, ***p < 0.001, **p < 0.01, *p < 0.05.

Almost all human complex traits and disease phenotypes exhibit some degree of sex differences, including variations in prevalence, age of onset, severity, and disease progression (Xia et al. 2024). The study of sex differences can help us determine the best treatment strategies for each sex for health conditions that exhibit sex differences. Therefore, we performed sex‐stratified analysis of differential disease expression for 13 NTS (Figure 8A). Sex differences of NTS, manifested in the direction and abundance of change, appear across all diseases and cell types (Figure 8A). For example, NTS is predominantly elevated in neurons of female with SCZ and predominantly decreased in males.

As shown in Figure 8B, red represents an increase in NTS in both male and female patients, blue represents a decrease in both, and white represents the opposite change. In terms of the direction of change, MDD showed the most significant gender difference, followed by ASD, SCZ, DS, MS, COVID‐19, and AD. In terms of expression abundance, most NTS showed significant gender expression differences except Endo and Peri. We found significant sex differences on glycine and GABA in the ExN of most diseases. Dopamine did not differ significantly in ExN, MOL, Endo, and Peri across all diseases. Norepinephrine did not differ significantly among InN, Astro, Micro, or Endo. There was no significant difference for serotonin in MOL, OPC, Endo, and Peri. There was no significant difference for GABA in MOL, Astro, and Endo.

### 2.8 NTS as a Biomarker for Disease Diagnosis

**FIGURE 9:**
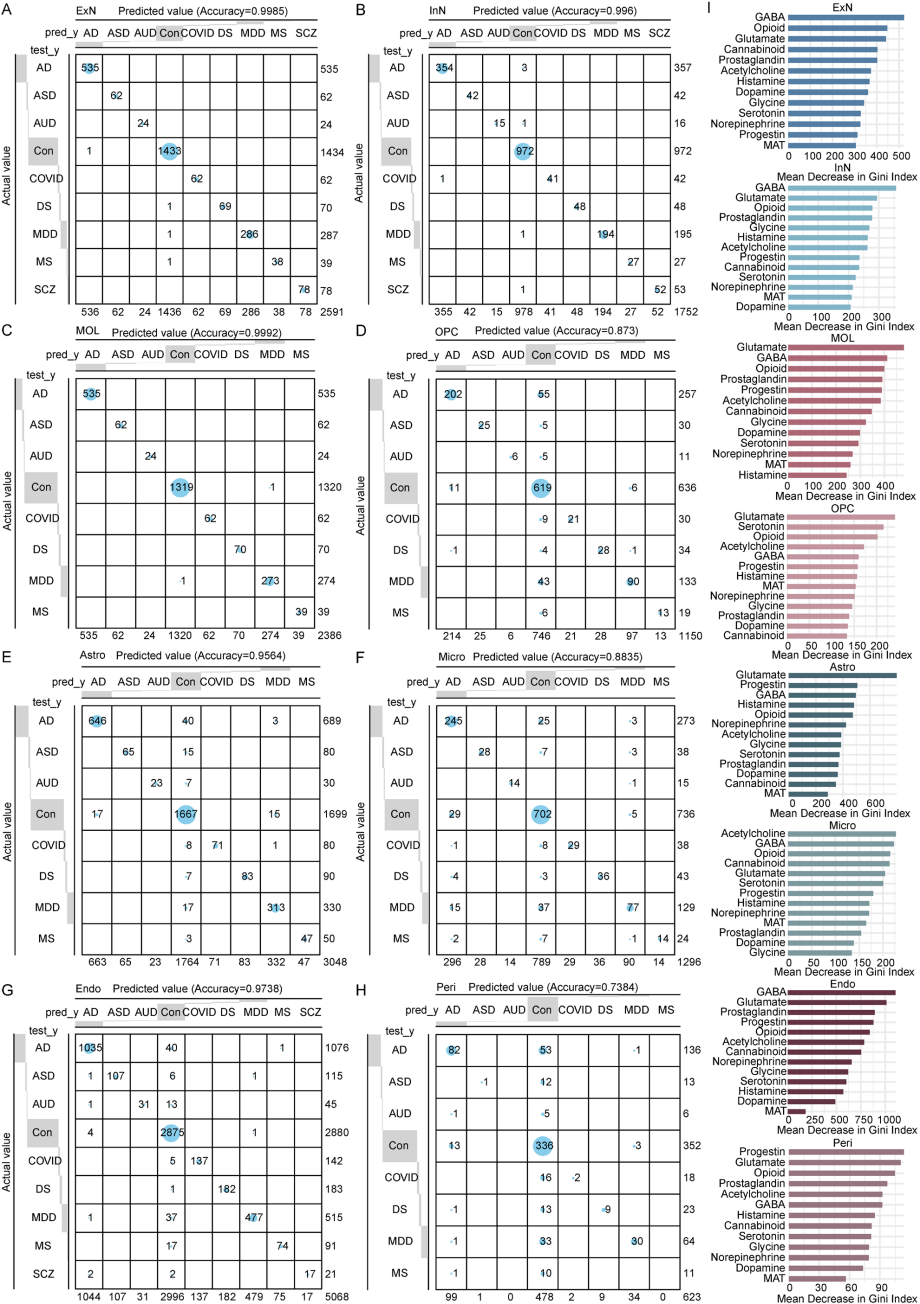
Within‐dataset benchmarking and feature‐importance analysis of NTS‐based disease discrimination across cell types. (A–H) Confusion matrices showing the within‐dataset discriminatory performance of NTS‐based random forest models for ExN, InN, MOL, OPC, Astro, Micro, Endo, and Peri. Accuracy values are shown for each cell type. (I) Feature‐importance analysis showing the mean decrease in Gini index for each NTS feature, highlighting their relative contributions to model discrimination across cell types.

Given the differential expression patterns of NTS across diseases, we next asked whether overall NTS score profiles could discriminate disease states in a predictive framework. We first report cell‐level benchmarks as an atlas‐enabled proof‐of‐concept use case, noting that cell‐level train/test splitting can permit donor‐level information leakage and that several disease‐versus‐control tasks are class‐imbalanced; therefore, these results should be interpreted as within‐dataset benchmarks rather than estimates of subject‐level generalizability. To obtain robust internal benchmarks, cells from each disease group were repeatedly resampled (30–50 times) and used as input for machine‐learning analyses. We constructed sample × NTS matrices and compared seven machine‐learning algorithms, including XGBoost, random forest, support vector machine (SVM), naïve Bayes, decision tree, nearest neighbor, and neural network models (Figure S7). Based on performance across these benchmarks, random forest showed the most consistent results and was selected for subsequent model interpretation (Figure S7). We then established cell‐type‐specific random forest models, in which NTS features in ExN, InN, and MOL showed relatively stronger discriminatory performance than those in other cell types (Figure 9A–H). Variable‐importance analysis further indicated that GABAergic, glutamatergic, and opioid‐related features contributed prominently to the models (Figure 9I), prioritizing these neurotransmitter systems for downstream investigation. To directly address potential donor‐level leakage and age‐related confounding, we additionally performed a SampleID‐aware evaluation in which donors were confined to a single split, disease‐specific age‐matched controls were used, and Age was excluded from predictive features. The resulting normalized confusion matrices and performance summaries are provided in Figuress S8 and S9 and Table S5. Given class imbalance, we report balanced accuracy and AUPRC, together with Cohen's kappa, to provide a more informative assessment than accuracy alone.

To further improve biological interpretability and reduce potential noise introduced by random resampling, we next used the scTenifoldKnk algorithm (Osorio et al. 2022), a virtual gene knockout method based on snRNA‐seq data, to identify regulatory genes associated with significantly altered NTS‐related genes. Gene modules composed of differential NTS genes and related regulatory genes are defined as disease‐related modules (Table S6). Seventy‐three genes (such as *ACTB*, *SLC17A7*) are regulated by NTS in at least seven disease states, suggesting that these genes have a broad role in neuropsychiatric disorders (Figure S10A). The PPI network map shows that genes such as *ACTB*, *CALM3*, *SLC17A7*, and *GRIA4* occupy a central position in the network, indicating their important role in multiple diseases (Figure S10B). *ACTB* and *SLC17A7* were the most shared genes (Figure S10C). SynGO analysis revealed that different disease module genes were involved in different synaptic dysfunctions (Figure S11). In various diseases, the biological processes that are significantly enriched are mainly concentrated in presynaptic and postsynaptic categories, and these categories show strong enrichment signals in several diseases (Figure S11). The number of bioprocess and synaptic genes associated with COVID‐19 was the highest, followed by MS and SCZ (Figure S11).

We then aimed to understand the role of module genes in defining disease states. To this end, we tested whether the expression of module genes could discriminate normal and diseased cells using automated machine learning models with training, validation, and test splits. The best‐performing classifier was then used for model interpretation, and permutation importance was applied to identify influential features. To further assess robustness, we additionally examined these disease‐associated module signals in independent external datasets, including the Mathys et al. brain dataset (*n* = 427) and disease‐related cerebral organoid datasets for AD, SCZ, DS, and ASD. These analyses provided supportive evidence that module‐gene‐based signals were reproducible across independent datasets, although their predictive performance varied by disease type (Figure S11). We further observed that the module‐related genes contributing to cell‐state discrimination differed across diseases (Figure S12). Models based on module‐gene composition showed stronger discriminatory ability in ASD, MDD, and SCZ, whereas performance was more limited in AD, AUD, COVID, MS, and DS (Figure S12). For example, the long noncoding RNA *MEG3* ranked among the most important features for distinguishing neuronal disease states in AD, COVID, and MDD (Figure S12A,D,G). Consistent with this finding, previous studies have shown that *MEG3* is upregulated in neurons from patients with AD and can induce neuronal necroptosis (Balusu et al. 2023). These results should therefore be interpreted as supportive cross‐dataset validation rather than definitive evidence of clinical generalizability.

## 3 Discussion

In brain aging and psychiatric disorders, the function of various neurotransmitter receptors and transporters is abnormally regulated, potentially impacting cognitive abilities, emotional regulation, and overall nervous system health (Chantranupong et al. 2023; Chen et al. 2024; Koh et al. 2023; Loerch et al. 2008). Integrating techniques such as single‐cell RNA sequencing, spatial transcriptomics, and proteomics allows for a detailed analysis of expression changes in neurotransmitter receptors and transporters within individual neurons or glial cells. Such integrated studies help reveal the specific roles these receptors and transporters play in neural networks, particularly in understanding how their expression in different immune cells (such as microglia) and neuronal subtypes influences aging and disease processes. Precise analysis of these cell types could uncover the mechanisms regulating specific pathways in neurodegenerative diseases and brain aging. This study provides a comprehensive assessment of the roles of 13 NTS in various neuropsychiatric disorders, highlighting cell‐type‐specific differences in NTS expression. The inclusion of multiple disease categories enabled us to interpret NTS alterations across distinct pathological contexts. AD primarily informed the aging‐related and neurodegenerative dimension of NTS dysregulation; DS highlighted developmental disturbance together with partial overlap with AD‐related signatures; ASD and SCZ emphasized psychiatric and neurodevelopmental heterogeneity; and COVID‐19 provided a contrasting neuroinflammatory context. This design allowed us to identify both shared NTS changes across diseases and disease‐specific deviations that may underlie distinct clinical and biological features. By integrating large‐scale snRNA‐seq datasets, this work offers an unprecedented perspective on NTS transformations in disease. We also provide an interactive platform for researchers to freely explore changes in NTS‐related gene expression across aging and disease.

This large‐scale data integration, compared to previous small‐sample studies focusing on single genes, provides a more comprehensive view of the molecular characteristics of NTS across different pathological states. For example, the observed upregulation of prostaglandins in microglia in Alzheimer's disease (AD) and multiple sclerosis (MS) suggests a potential role in promoting microglial activation and neuroinflammatory responses (Minhas et al. 2021; Yin et al. 2022; Serrats et al. 2017). This provides new clues for exploring the pathogenesis of AD and MS and suggests that the prostaglandin pathway may be an important target for intervention in neuroinflammatory and neurodegenerative diseases. Consistent with previous studies, *GRIA1* mRNA was significantly elevated in pyramidal neurons of DLPFC in patients with SCZ (O'Connor and Hemby 2007). We found that *GRIA1* expression was significantly upregulated in ExN and InN across all diseases. The role of *GRIA1* in the nervous system is dual‐sided. *GRIA1* upregulation is often associated with enhanced long‐term potentiation (LTP), a key synaptic mechanism involved in learning and memory processes (Schmitt et al. 2005; Xu et al. 2020; Sanderson et al. 2016). However, sustained upregulation of *GRIA1* expression keeps neurons in a prolonged hyperexcitable state, increasing the risk of neurotoxicity and excitotoxicity (Ibanez et al. 2022). This sustained high level of excitability may lead to calcium ion overload in neurons, subsequently inducing apoptosis and neuronal damage (Esteras et al. 2022; Zlokovic 2008). In patients with schizophrenia, excessive *GRIA1* expression may increase the risk of positive symptoms, such as hallucinations and delusions, due to abnormal neuronal excitation from overactivity in the glutamate system (Greger et al. 2017; Shaffer et al. 2013). During manic episodes of bipolar disorder, *GRIA1* upregulation may lead to excessive synaptic excitability, placing the patient in a highly activated emotional state (Parekh et al. 2018). In neurodegenerative diseases such as AD, *GRIA1* upregulation may worsen neuroinflammation and cell damage (Esteras et al. 2022). Genome‐wide Mendelian randomization identified *GRIA1* as a novel drug target for BPD (Liu et al. 2023). Studies have shown that a decrease of *GRIA1* in multiple hippocampal subregions impairs spatial working memory. Transgene‐mediated *GRIA1* ablation in CA2 excitatory cells impairs short‐term memory for homogenous objects. *GRIA1* gene knockout in CA3 pyramidal cells mildly impairs object‐related and spatial short‐term memory but appears to partially increase social interaction, enhance sustained attention, and reduce motor impulsivity (Kilonzo et al. 2022). Moreover, the upregulation of hippocampal *GRIA1* may be related to hypermotor activity in older rodents and may serve as a therapeutic target for hypertensive irritability and hyperactivity (Yen et al. 2022). *GRIA1* is associated with the development and recognition of astrocytes (Xu et al. 2021). Astrocytes utilize *GluA1* to clear glutamate from the synaptic gap, thereby preventing excitotoxicity (Seifert et al. 2006). *GRIA1* activation enhances astrocytes' ability to sense changes in glutamate concentrations, enabling them to respond more rapidly to nerve damage, ischemia, or other pathological conditions (Miao et al. 2023). Therefore, achieving a therapeutic effect across the broad spectrum of disease symptoms may require carefully balancing *GriA1*‐mediated *AMPAR* signaling. A recent microarray dataset study showed that *CHRM3* is inversely associated with age in normal brain tissue and is significantly reduced in AD (Sanfilippo et al. 2023). However, we found that *CHRM3* is significantly upregulated in ExN and InN cells in various diseases, including AD, and its expression is positively correlated with age in normal brain tissue but inversely correlated in glial cells. The *CHRM3* gene encodes the M3 muscarinic acetylcholine receptor (M3 receptor), which primarily acts on postsynaptic acetylcholine signaling pathways, regulating neuronal excitability, synaptic plasticity, and various brain functions (Ye et al. 2024; Smith et al. 2024; Zheng et al. 2023). However, excessive *CHRM3* expression or activity may lead to neuronal overexcitation, resulting in positive symptoms such as hallucinations and delusions. Chronic overactivation of *CHRM3* may trigger intracellular calcium overload, causing neurotoxicity and further damage to synapses and neurons (Ferreira‐Vieira et al. 2016; Wess et al. 2007). This phenomenon is especially evident in the advanced stages of AD, where *CHRM3* overactivity may lead to excitotoxicity and exacerbate neurodegeneration (Ferreira‐Vieira et al. 2016). However, it remains unclear whether the elevation of *GRIA1* and *CHRM3* in aging and disease is compensatory or pathogenic. Recent studies have shown that the muscarinic acetylcholine receptor (mAChR) agonist xanomeline can reduce psychotic symptoms and improve cognition in patients with AD and SCZ, suggesting that elevated *CHRM3* may serve as a compensatory mechanism (Paul et al. 2022, 2024; Tobin 2024).

The study also offers important insights into NTS dynamics during aging, with significant fluctuations observed in the fifth decade of life—a critical period of increased risk for neurodegenerative diseases (Colantuoni et al. 2011; Lu et al. 2004). Age‐related fluctuations in NTS may reflect increased diversity in neurotransmitter signal transduction and regulation, placing the brain under greater stress as it adapts to aging. We cannot directly observe how NTS changes with age in patients with various psychiatric disorders. However, by analyzing age‐related NTS changes in disease, we can identify which changes accelerate or worsen specific psychiatric symptoms. Recognizing these age‐related NTS changes could further illuminate the link between aging and neuropsychiatric disorders, offering a critical window for early intervention. For example, dynamic changes in specific receptors and transporters in the PFC could serve as predictive markers of aging‐related diseases, guiding directions for preventive treatment (Lu et al. 2004). Additionally, we observed plasticity and compensatory mechanisms in the NTS system, where two functionally similar receptors or transporters from the same or different systems exhibit opposite expression patterns in aging and disease states. In these conditions, the nervous system attempts to compensate for damaged neural networks by upregulating or downregulating receptor or transporter expression.

Utilizing spatial transcriptome data, we observed that genes associated with various neurotransmitter systems display distinct spatial distribution patterns across the hierarchical layers of the cerebral cortex. Notably, genes linked to excitatory and inhibitory neurotransmitters, such as glutamate and GABA, are predominantly concentrated in the intermediate layers (L3–L5), which may serve as critical regions for information integration (Harris and Shepherd 2015). Genes associated with neurotransmitters, such as dopamine and serotonin, are predominantly found in the deeper cortical layers (L5–L6) and WM. This distribution suggests their crucial roles in cortical output and neural regulation (Doya 2008; Shepherd 2013; Celada et al. 2013). Moreover, the spatial distribution of myelin and metabolism‐related genes, such as the *PAQR* family and *SLC30A1*, underscores the role of white matter in providing neural support and facilitating signal transmission (Schumacher et al. 2012). The specific enrichment of progestin in WM indicates its significant role in myelin‐related diseases, including AD and MS (Chen et al. 2022; Luo et al. 2022; Bencker et al. 2024). This study elucidates the spatial specificity of gene expression, offering a novel perspective for comprehending the pathological mechanisms underlying brain functions and neurotransmitter systems. The findings provide valuable insights into the spatial organization and functionality of neurotransmitter systems, while also establishing a spatial context for investigating disease‐associated genes, such as those implicated in neurodegenerative conditions.

Amphoteric dimorphism occurs in a wide range of neurotransmitter systems, including serotonin, GABA, acetylcholine, vasopressin, opioids, and monoamines (Cahill 2006; McEwen and Milner 2017). Studies have shown that women report significantly higher levels of monoamine oxidase in several regions of the brain compared to men. Additionally, sex differences are evident in the rate of serotonin synthesis in the brains of healthy individuals, and the levels of serotonin metabolites in postmortem tissues (Cahill 2006). The sex difference of NTS in the disease is related to the clinical phenotype and drug response of patients (Soni et al. 2024). This study reveals the heterogeneity of NTS between sexes, highlighting the significant influence of sex on disease‐related changes in NTS. The study showed a significant decrease in glutamate levels in the brains of male AD mice, whereas female mice exhibited little change (Soni et al. 2024). We found that glutamate‐related receptors and transporters varied significantly more in ExN and InN cells in male AD patients compared to female patients. Additionally, studies have shown that the male‐to‐female ratio for ASD diagnosis is approximately 3:1 (Loomes et al. 2017). We found that NTS differences in ExN cells were more significant and prevalent in men with ASD, while they were weaker in women. Gender differences in NTS composition and regulatory networks may have important implications for drug response and disease progression. The discovery of sex‐specific NTS differences is expected to enhance personalized treatment strategies for neuropsychiatric disorders, particularly for those with clear gender differences, such as major depression and autism spectrum disorders (Maitra et al. 2023). Future studies will require more experimental data on sex differences to validate these findings and explore the molecular mechanisms by which sex factors regulate NTS.

An important contribution of this study is the establishment of a multi‐cell type expression map of NTS, revealing that NTS is significantly expressed not only in neurons but also has unique functions in glial and cerebrovascular cells. This finding underscores that neurotransmitter systems play roles beyond neuronal regulation, significantly impacting supportive brain cells like microglia and astrocytes. Brain endothelial cells express a variety of amino acid (AA) transporters, such as glutamate and aspartic acid, providing a mechanism for the net removal of potentially neurotoxic AA from the brain (Zlokovic 2008). The NTS expression pattern in cerebrovascular cells further suggests a potential role for blood–brain barrier function in disease development. This multi‐cell type perspective offers new insights into the systematic analysis of NTS in diseases, especially in considering brain supportive cells as therapeutic targets, providing a foundation for comprehensive disease treatment.

Finally, by constructing modules of NTS and their regulatory genes, this study provides candidate features that may help distinguish healthy from pathological states and offers a resource for feature prioritization, disease modeling, and future mechanistic investigation. Screening disease‐related NTS networks suggests that these modules could serve as a reliable basis for future drug screening and intervention studies. NTS module genes in a variety of diseases are involved in pre‐ and postsynaptic function, underscoring the widespread role of synaptic dysfunction in these diseases. We found that core genes such as *ACTB* and *SLC17A7* are shared across multiple diseases. A limitation of the machine‐learning analyses is that predictive performance was evaluated primarily within integrated datasets and may still be influenced by donor number, class imbalance, and interindividual heterogeneity; therefore, these results are best interpreted as exploratory feature‐prioritization analyses rather than robust clinically generalizable classifiers.

Research has demonstrated that the *SLC17A7* gene encodes vesicular glutamate transporter 1 (*VGLUT1*), which is expressed in central monoamine, acetylcholine, and GABA neurons, as well as in primary glutamate‐responsive neurons (El Mestikawy et al. 2011). This finding underscores the complexity of morphological and functional diversity within the neuronal system characterized by dual signaling capabilities. Furthermore, it suggests that additional vesicular interactions may be involved beyond the glutamate transport function attributed to *SLC17A7*. The activation of glutamate‐responsive pathways associated with *VGLUT1* expression has been linked to psychological stress. The activation of the *VGLUT1*‐positive glutamatergic pathway has been linked to psychological stress (Kataoka et al. 2020). Prolonged or excessive exposure to stress may result in disruptions within neurobiological and neuroimmunological systems, thereby heightening vulnerability to a range of neuropsychiatric disorders, including depression, anxiety, SCZ, and BPD. Additionally, analyses of disease‐related brain organoids revealed similarities in NTS composition between certain organoids and patient brain tissue, indicating that organoids could model disease‐specific NTS networks—an invaluable resource for drug development and disease modeling. These advances lay the foundation for precise treatment of various neuropsychiatric disorders, underscore the importance of NTS in disease modeling, and provide new directions for understanding the complex mechanisms of neurological diseases.

However, more in‐depth studies are necessary. (1) From Mechanism to Intervention: Translational research is needed to understand how antidepressants, antipsychotics, and neuroprotectants regulate receptor and transporter function, particularly in the context of aging and various diseases. The effects of non‐pharmacological interventions, such as electrical stimulation and cognitive training, on these pathways also warrant investigation. (2) Exploring Novel Therapeutic Targets and Approaches: Are there new receptors or transporters specific to certain mental disorders and brain aging that could serve as drug targets? What role could gene‐editing technologies like CRISPR play in regulating receptor and transporter expression (Mountoufaris et al. 2024; Soden et al. 2023)? Applying these emerging technologies to neurotransmitter regulation could open new pathways for precision therapies. (3) Real‐Time Imaging of Dynamic Receptor and Transporter Changes: Technologies such as positron emission tomography (PET) and functional magnetic resonance imaging (fMRI) allow for real‐time observation of receptor and transporter distribution and function in the brain, enhancing our understanding of their roles across aging stages and disease contexts (Hansen et al. 2022). (4) Cross‐Species Studies: Comparing receptor and transporter expression patterns and regulatory mechanisms between humans and model animals can accelerate the identification of precise therapeutic targets.

## 4 Methods

### 4.1 Data Download and Collection

Through a literature review, we collected and organized snRNA‐seq data from the human PFC covering eight diseases across 17 datasets (Table S1). The database includes Gene Expression Omnibus (GEO), synapse, ArrayExpress, European Nucleotide Archive (ENA), European Genome‐phenome Archive (EGA), and National Genomics Data Center (NGDC). Details of the samples, including the database, sample ID, age, gender, and diagnosis, can be found in Table S1. Each dataset included normal individuals who underwent strict clinical assessments to ensure they had no active neuropsychiatric disorders. Individuals with each disease were diagnosed by three clinical practitioners. Informed consent was obtained from each participant. They also signed the Anatomy Gift Act and a repository consent form, allowing their data to be reused.

### 4.2 Pre‐Processing and Quality Control of snRNA‐Seq Data

In addition to SCZ data, all raw sequencing data were processed using Cell Ranger 7.0.1 (10× Genomics) to obtain expression matrices for downstream analysis. Raw snRNA‐seq count matrices are available at https://doi.org/10.5281/zenodo.6921620. To ensure dataset quality, we retained only cells with more than 200 detected genes and < 5% mitochondrial gene content. Doublets were detected using DoubletFinder (v2.0.3). After sample integration and clustering, clusters lacking specific marker genes, with relatively low gene content, and high mitochondrial ratios were discarded.

### 4.3 De‐Batch Integration of Multiple Datasets

We integrated the scRNA‐seq data of healthy and disease individuals separately to remove batch effects. Briefly, unique molecular identifiers (UMIs) from each valid cell barcode were retained for all downstream analyses and processed using the Seurat R package (v4.2.2). We first used reciprocal PCA (RPCA) instead of CCA to identify an effective space for finding anchors. When using RPCA to determine anchors between any two datasets, each dataset was projected into the PCA space of the other, and anchors were constrained by requiring mutual nearest neighbors. For batch integration, individuals aged < 60 years were defined as adult, whereas those aged ≥ 60 years were defined as elderly. We randomly designated two samples each from adult males, adult females, elderly males, elderly females, females with disease, and males with disease as the “reference” dataset for integration analysis, while the remaining datasets were designated as the “query” datasets. We also used the *FindIntegrationAnchors* and *IntegrateData* functions to remove batch effects, followed by clustering analysis using the *FindNeighbors* and *FindClusters* functions. To evaluate the quality of batch correction, we quantified both batch mixing and biological conservation after integration. Batch mixing was assessed using LISI_batch_mean, LISI_batch_median, batch_entropy_mean, and batch_entropy_norm_mean, whereas biological conservation was evaluated using ASW_celltype and celltype_purity_knn_mean. In addition, integrated embeddings were visually inspected by coloring cells according to dataset and cell type. Data visualization was performed using nonlinear dimensionality reduction methods such as UMAP and t‐SNE.

### 4.4 Cell Type Identification

Cell type annotation was performed using the method provided by the SingleR package, which identifies cell types based on reference datasets. This method annotates the cells to be identified as the cell type that has the highest correlation with the single‐cell reference expression profile dataset. The results of the dataset identification in this report are provided for reference, and further descriptions and validations of the cell populations will be made based on relevant genes from existing literature. The detailed markers for the main cell types are as follows: Neuron: *RBFOX1*, *SNAP25*, and *SYT1*; ExN: *SLC17A* and *CAMK2A*; InN: *GAD1* and *GAD2*; MOL: *PLP1* and MBP; OPC: *OLIG1* and *OLIG2*; Astro: *GFAP* and *AQP4*; Micro: *CSF1R* and *CD74*; Endo: *FLT1* and *CLDN5*; Peri: *DCN* and *COL1A2*.

### 4.5 Integration of Data From the Same Cell Type Across Different Datasets

SnRNA‐seq data of each cell type from Control and disease group were separately extracted and merged. First, we used the *SCTransform* function to standardize the data, followed by PCA dimensionality reduction using the *RunPCA* function. We then performed batch integration using the *RunHarmony* function with the specific parameters: group.by.vars = “SampleID,” assay.use = “SCT,” max.iter.harmony = 30. Next, we conducted clustering analysis using the *FindNeighbors* and *FindClusters* functions, and visualized the data using UMAP.

### 4.6 Gene Ontology (GO) Enrichment Analysis

The *enrichGO* function from the clusterProfiler R package was used for enrichment analysis, employing the Benjamini–Hochberg (BH) method for multiple test correction. A GO term with an adjusted *p* value < 0.05 was considered significantly enriched. The *enrichmentNetwork* function from the aPEAR package was used for the classification of enriched terms and the construction of the network.

### 4.7 Gene Set Score Analysis

Gene set scores were obtained by analyzing the transcriptome of each input cell against the aforementioned gene sets using the Seurat function *AddModuleScore*. Changes in scores between groups were analyzed with the ggpubr R package using the Wilcoxon test.

### 4.8 Identification of Differentially Expressed Genes (DEGs)

To identify genes that are differentially expressed in aging or disease, *p* values were calculated and FDR‐corrected using the MAST method (Schirmer et al. 2019). All nuclei from different sample groups corresponding to specific cell types were included. MAST was utilized to perform zero‐inflated regression analysis by fitting a linear mixed model. To account for confounding factors such as age, sex, and the proportions of ribosomal and mitochondrial transcripts, the following model for aging and disease was fitted using MAST:

$$\mathrm{zlm}\left(\sim \text{condition}+\text{nCount}\_\mathrm{RNA}+\text{percent}.\mathrm{mt}+\mathrm{Sex},\mathrm{sca},\text{method}=\text{glmer},\text{ebayes}=T\right)$$

$$\mathrm{zlm}\left(\sim \text{condition}+\text{nCount}\_\mathrm{RNA}+\text{percent}.\mathrm{mt}+\mathrm{Sex}+\mathrm{Age},\mathrm{sca},\text{method}=\text{glmer},\text{ebayes}=T\right)$$

To identify DEGs due to age or disease effects, a likelihood ratio test was performed by comparing models with and without the diagnostic factor. Genes exhibiting at least a 25% increase or decrease in expression between groups, along with a false discovery rate (FDR)‐corrected *p* value of < 0.05, were selected as differentially expressed.

### 4.9 Expression‐Weighted Cell‐Type Enrichment Analysis

Expression‐weighted cell‐type enrichment (EWCE) (Zhu et al. 2021; Skene and Grant 2016) was employed for the analysis of disease gene enrichment using default parameters. This analysis was conducted separately for each species dataset. This analysis was performed separately for each species dataset. To mitigate the bias effect, we initially employ the “vst()” function to conduct a variance‐stabilizing transformation on the unique molecular identifier count matrix. Subsequently, we utilize the “fix_bad_mgi_symbols()” function to rectify gene nomenclature. The “drop_uninformative_genes()” function is then applied to eliminate non‐informative genes, thereby reducing computational time and minimizing noise in subsequent analyses. Following this, we implement the “generate.celltype.data()” function to compute the specificity matrix for each dataset. We perform 100 bootstrap resamplings with replacement on all detected genes within each species dataset as a background reference, and *p* values are adjusted using the Benjamini–Hochberg method. A significance threshold of 0.05 is established.

### 4.10 Comparative Analysis of Organoids and Parental Data

Organoid datasets from AD, ASD, BPD, SCZ, and DS were subjected to integration and subsequent cell type identification. In summary, the cell gene expression matrix was log‐normalized, and the *FindVariableFeatures* function was employed to identify 3000 highly variable genes exhibiting substantial intercellular variability. The *FindIntegrationAnchors* and *IntegrateData* functions were utilized for canonical correction analysis across individual samples. Following the removal of batch effects while preserving the biological variability inherent in the dataset, clustering analysis was conducted using the *FindNeighbors* and *FindClusters* functions. UMAP was employed for data visualization. Following cell type identification, NTS gene set scores were computed by the *AddModuleScore* function, and intergroup comparisons were conducted for the various disease cell types.

### 4.11 Disease Prediction of Cell Type Using Machine Learning (ML)

To assess whether aggregate NTS features carried disease‐discriminative information at the cell level, we evaluated seven machine‐learning algorithms, including XGBoost, random forest, support vector machine (SVM), naïve Bayes, decision tree, k‐nearest neighbors, and neural network models. For the within‐dataset benchmarking analysis, 25% of nuclei were assigned to the test set, and the remaining 75% were used for training and validation. Model performance was compared using accuracy and ROC‐AUC, and the best‐performing algorithm was selected for subsequent interpretation. Hyperparameters were optimized using repeated 10‐fold cross‐validation. These analyses were intended as exploratory within‐dataset assessments of disease‐discriminative NTS features rather than definitive evidence of robust subject‐level or clinical generalizability.

Another approach involves constructing predictive models based on the expression levels of NTS module genes within the cells. The snRNA‐seq data corresponding to specific cell types were selected for the classification of control versus disease states. The pipeline and functionalities were implemented using the automated machine learning R package h2o (v3.44.0.2). For data partitioning, 50% of the nuclei were designated for the test set, while the remaining 50% were further divided into training and validation sets using 10‐fold cross‐validation and a maximum of 20 models. The optimal model was extracted using the function *h2o.get_best_model*. The function *h2o.predict* was applied to the test and validation sets. To assess variable importance, the function *h2o.permutation_importance_plot* was utilized based on permutation analysis.

### 4.12 Virtual Knockout of the Gene of Interest

To analyze the function of module gene knockout (KO) in specific cell types of particular diseases, we extracted disease‐ and cell‐specific snRNA‐seq data and used the gene × cell expression matrix as input for scTenifoldKnk (Osorio et al. 2022). The genes perturbed by the virtual knockout with FDR‐corrected *p* < 0.05 were selected as differentially expressed. Interaction enrichment analysis was conducted based on the STRING protein–protein interaction database (https://version‐12‐0.string‐db.org/). We selected biological processes (GO) and human phenotypes (Monarch) as our items of interest.

### 4.13 Acquisition of Immunohistochemical Data

To validate the results, we searched for the expression of proteins of interest in the Human Protein Atlas database (https://www.proteinatlas.org/). All antibodies have undergone rigorous validation for specificity, reproducibility, and functionality and have been tested in various experimental applications.

### 4.14 Statistical and Reproducibility

The statistical analyses were done in R (v4.2.2) if not specified. Data visualization is implemented using R, Prism10, Cytoscape, and Adobe Illustrator 2021. We state that no statistical method was used to predetermine sample size.

## Author Contributions

R.‐Z.N., X.‐Q.Z., and X.‐F.Z. conceptualized, acquired funding, and supervised this study. Data were processed, analyzed, and visualized by R.‐Z.N., M.‐Y.Z., Y.‐P.L., and J.W. The manuscript was drafted by R.‐Z.N., W.‐W.W., Y.‐J.W., and Y.R., and was reviewed and edited by T.‐H.B., X.‐F.Z., X.‐L.L., and Y.D. All authors discussed results and commented on the manuscript.

## Funding

This study was supported by National Natural Science Foundation of China (82501796, 82160269, 82360275, 82160272), Yunnan Provincial Department of Science and Technology Plan Project (202405AC350104, 2018FE001‐223, 202301AY070001‐020), Kunming Health Science and Technology Talent Training Project (Thousand project, 2024‐SW (Reserve)‐72; 2024‐SW (Reserve)‐73), Scientific Research Project of the Provincial Clinical Medical Center of Yunnan Province (2024YNLCYXZX0278, 2024YNLCYXZX0280), and Research Project on the Construction of Provincial‐Level Regional Medical Centers in Yunnan Province (HH‐202512‐005; HH‐202512‐006; HH‐202512‐007; HH‐202512‐008).

## Ethics Statement

The authors have nothing to report.

## Consent

The authors have nothing to report.

## Conflicts of Interest

The authors declare no conflicts of interest.

## Supporting information


**Figure S1:** Split UMAP visualization of the integrated human PFC snRNA‐seq atlas across aging and CNS disorders.
**Figure S2:** Subtype‐specific markers and cell proportions across different diseases.
**Figure S3:** Age‐associated clustering and expression dynamics of NTS‐related genes in excitatory and inhibitory neurons.
**Figure S4:** Age‐associated clustering and expression dynamics of NTS‐related genes in MOL, OPC, Astro, and Micro.
**Figure S5:** Comparison of interactions and neuronal subtype networks across diseases.
**Figure S6:** NTS‐related gene interaction networks in glial and OPCs across diseases.
**Figure S7:** Machine learning model performance in predicting cell disease states using NTS‐related genes.
**Figure S8:** Normalized confusion matrices for disease‐versus‐age‐matched control classification in ExN, InN, Astro, and Micro.
**Figure S9:** Normalized confusion matrices for disease‐versus‐age‐matched control classification in MOL, OPC, Endo, and Peri.
**Figure S10:** Cross‐disease analysis of NTS module‐related genes.
**Figure S11:** SynGO analysis of NTS module‐related genes in biological processes (BP) across diseases.
**Figure S12:** Prediction of disease states using NTS module‐related genes in ExN.

**Table S1:** Sample database source and basic information.
**Table S2:** The number of cell for cell type from different diseases.
**Table S3:** NTS‐related genes.
**Table S4:** NTS related differentially expressed genes in different disease.
**Table S5:** SampleID‐aware disease‐versus‐age‐matched control classification performance across major cell types.
**Table S6:** NTS module‐related genes for ExN in different diseases.

## Data Availability

The processed expression matrices used and/or analyzed during the current study and corresponding code for this research are available from the corresponding author on reasonable request.
