# Supplementary material for: Multi‐Omics Reveals Dysregulated Neurotransmitter Systems in Aging and CNS Disorders
Source: Aging Cell. 2026 May 13;25(5):e70544. doi: 10.1111/acel.70544 (PMC13171466; doi:10.1111/acel.70544)
Supplement: Supplementary file 1 — Figure S1: Split UMAP visualization of the integrated human PFC snRNA‐seq atlas across aging and CNS disorders. Figure S2: Subtype‐specific markers and cell proportions across different diseases. Figure S3: Age‐associated clustering and expression dynamics of NTS‐related genes in excitatory and inhibitory neurons. Figure S4: Age‐associated clustering and expression dynamics of NTS‐related genes in MOL, OPC, Astro, and Micro. Figure S5: Comparison of interactions and neuronal subtype networks across diseases. Figure S6: NTS‐related gene interaction networks in glial and OPCs across diseases. Figure S7: Machine learning model performance in predicting cell disease states using NTS‐related genes. Figure S8: Normalized confusion matrices for disease‐versus‐age‐matched control classification in ExN, InN, Astro, and Micro. Figure S9: Normalized confusion matrices for disease‐versus‐age‐matched control classification in MOL, OPC, Endo, and Peri. Figure S10: Cross‐disease analysis of NTS module‐related genes. Figure S11: SynGO analysis of NTS module‐related genes in biological processes (BP) across diseases. Figure S12: Prediction of disease states using NTS module‐related genes in ExN. [file ACEL-25-e70544-s002.docx]

**Supplementary Materials for**

**Multi-omics Reveals Dysregulated Neurotransmitter Systems in Aging and CNS Disorders**

*Niu et al.*

**This PDF file includes:**

Supplementary Figure 1 to 12

Captions of Tables S1 to S6

**
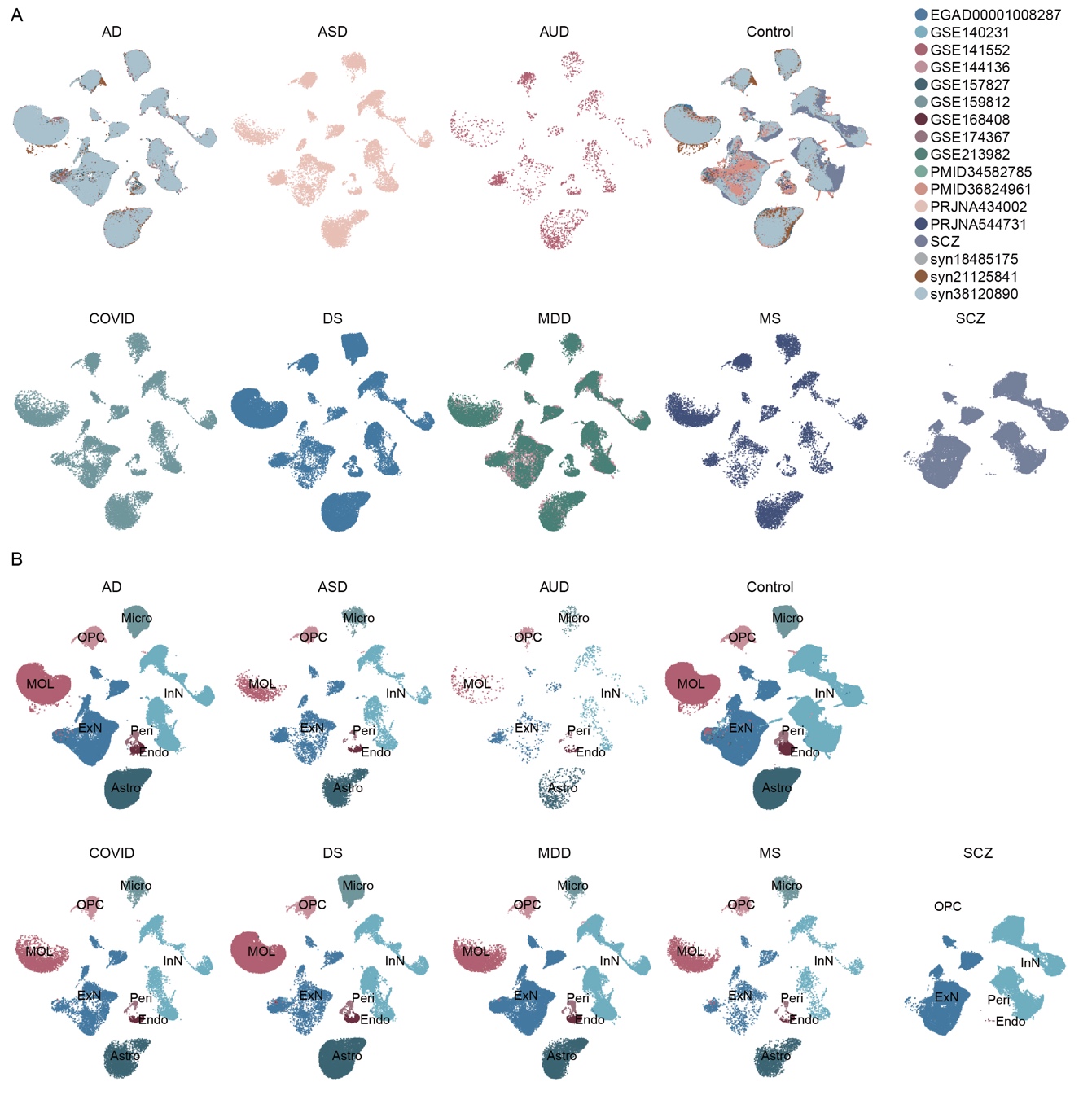
**

**Supplementary Figure. 1. Split UMAP visualization of the integrated human PFC snRNA-seq atlas across aging and CNS disorders.** **(A)** UMAPs split by disease group and colored by dataset, showing dataset-specific distributions after integration. **(B)** UMAPs split by disease group and colored by annotated major cell types, illustrating preservation of cell-type structure across conditions. These split views were generated to improve visualization of overlapping cells in the integrated embedding.

**
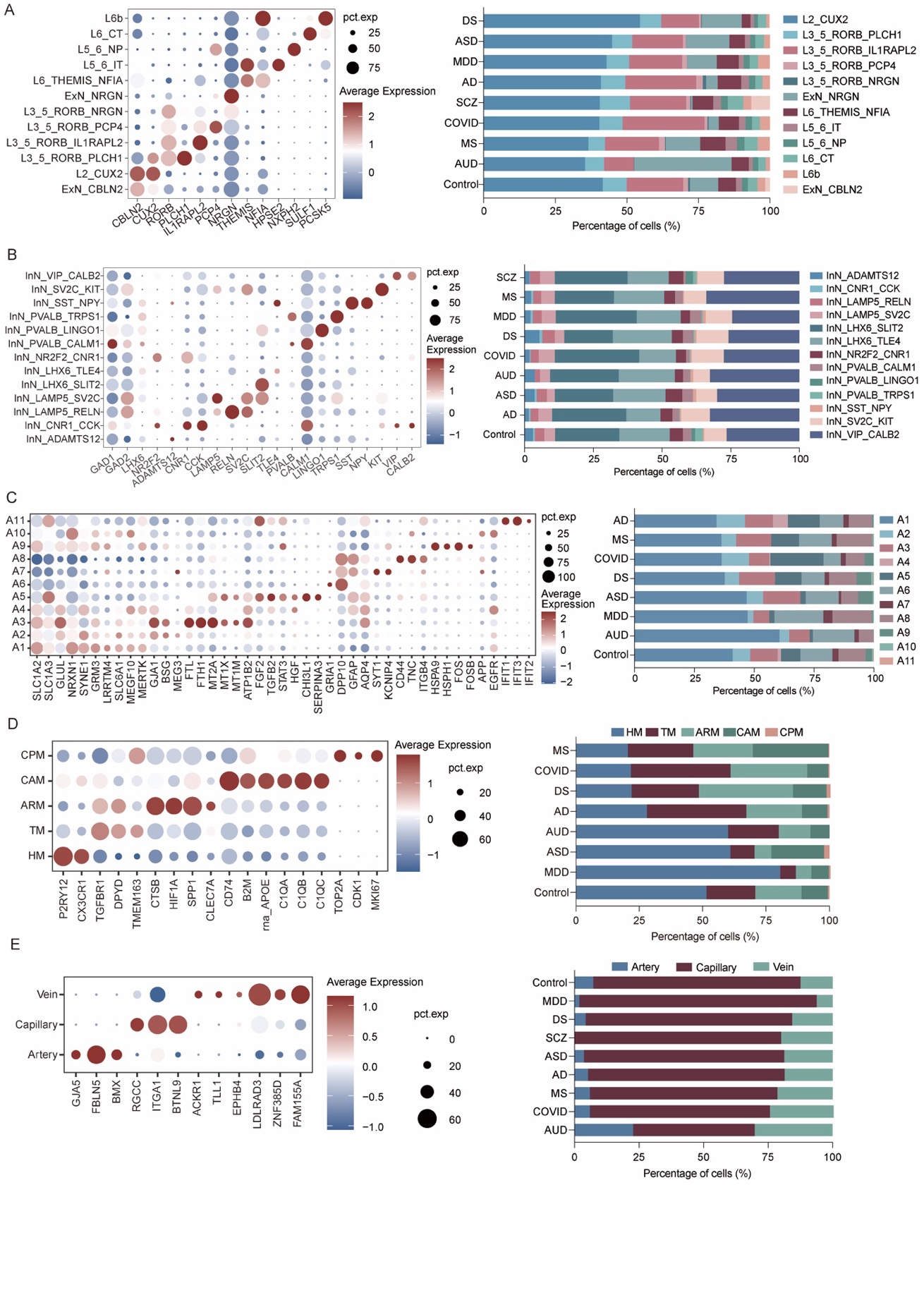
**

**Supplementary Figure. 2. Subtype-specific markers and cell proportions across different diseases.** Dot plot demonstrating subtype-specific marker expression patterns across cell subtypes in ExN (A), InN (B), Astro (C), Micro (D), and Endo (E). The color intensity represents average expression levels, while the dot size indicates the percentage of cells expressing the marker. Bar plots on the right indicate the proportions of these subtypes across different disease conditions.


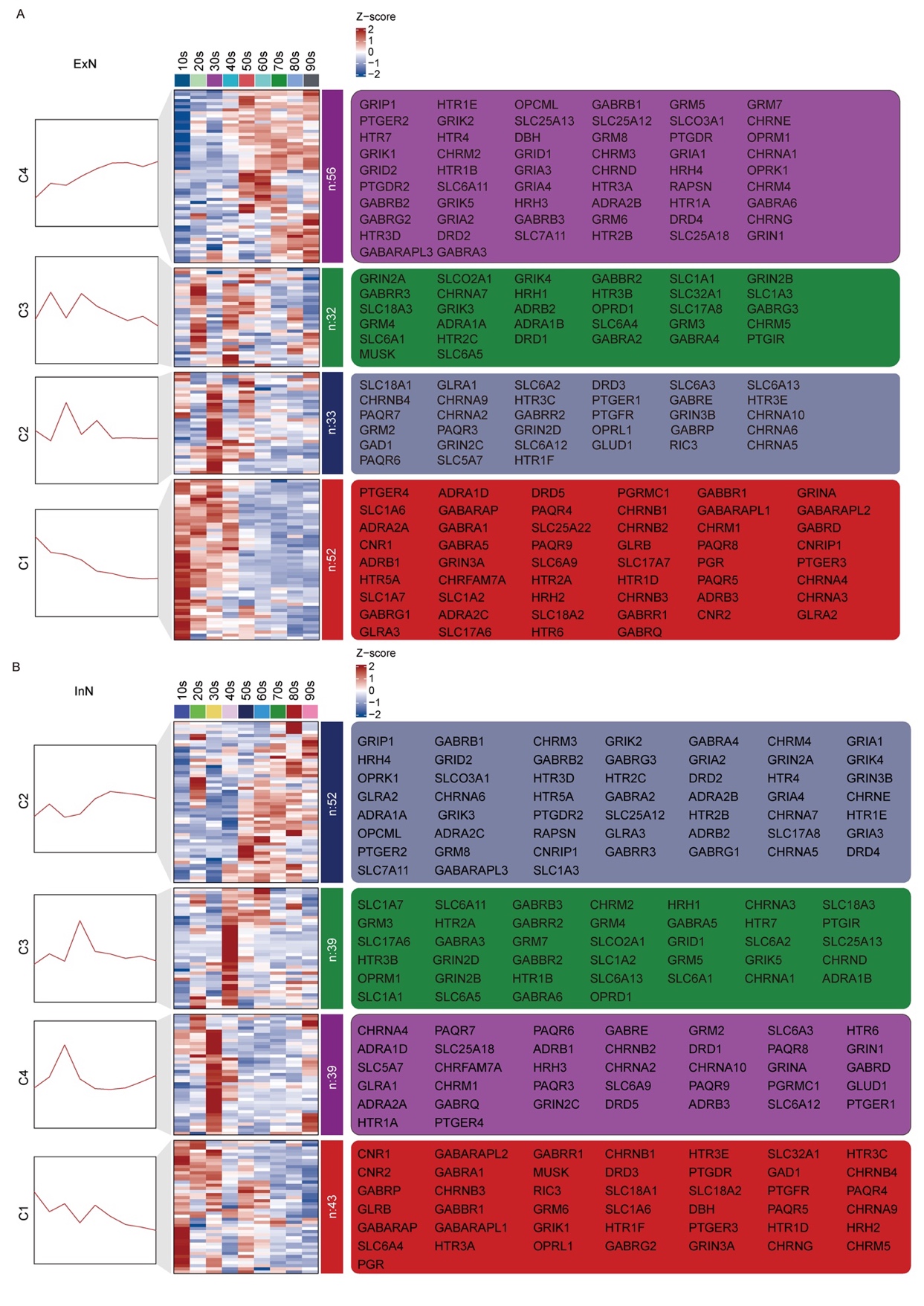


**Supplementary Figure. 3.** Age-associated clustering and expression dynamics of NTS-related genes in excitatory and inhibitory neurons. Heatmap showing the Z-scores of NTS-related gene expression in ExN (A) and InN (B) across different age groups (10s–90s). Genes are grouped into four distinct clusters (C1–C4) based on expression patterns. Line plots on the left illustrate the average expression trends of each cluster across ages.


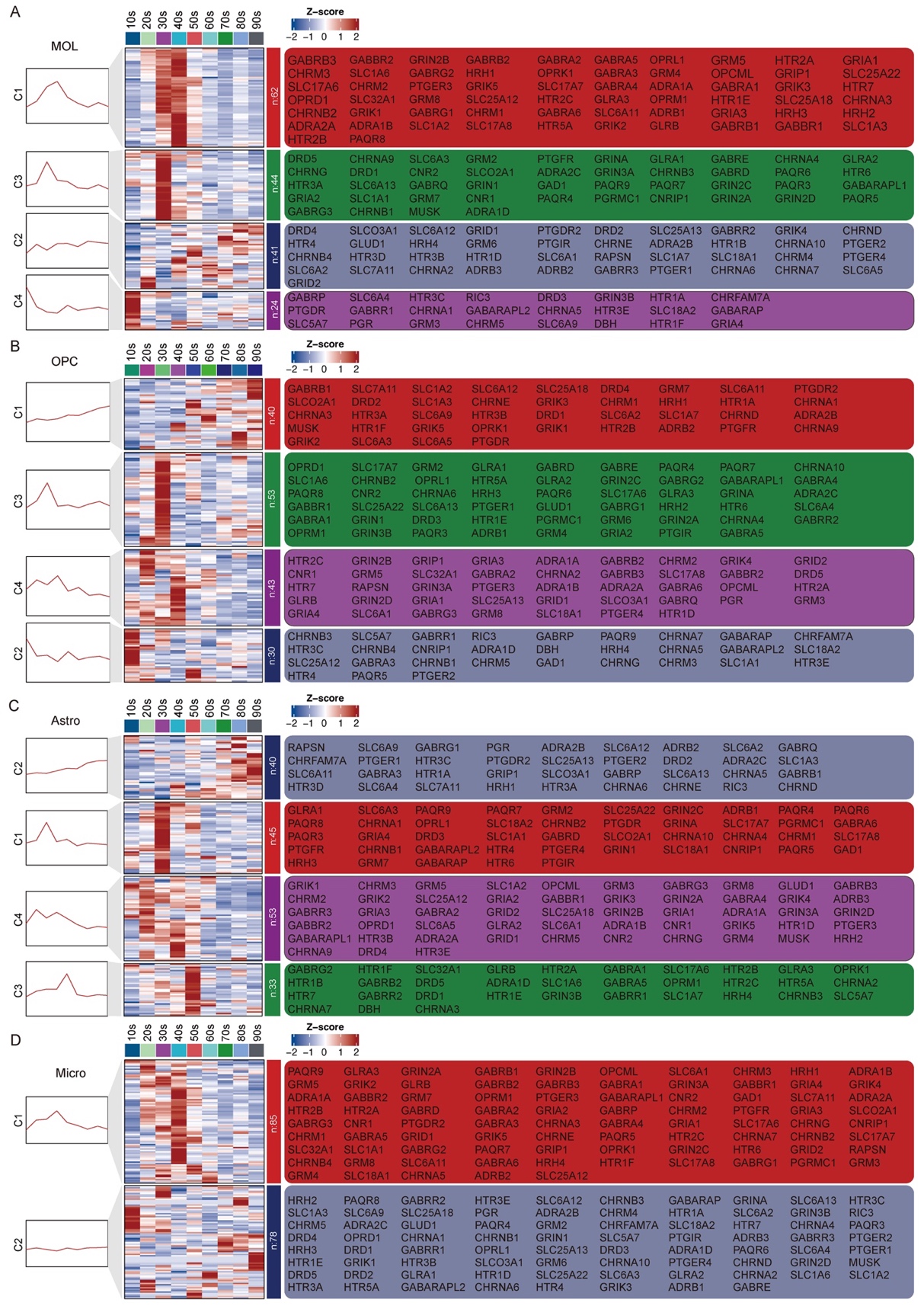


**Supplementary Figure. 4.** Age-associated clustering and expression dynamics of NTS-related genes in MOL, OPC, Astro, and Micro. Heatmap showing the Z-scores of NTS-related gene expression in MOL (A), OPC (B), Astro (C), and Micro (D) across different age groups (10s–90s). Genes are grouped into four distinct clusters (C1–C4) based on expression patterns. Line plots on the left illustrate the average expression trends of each cluster across ages.

**
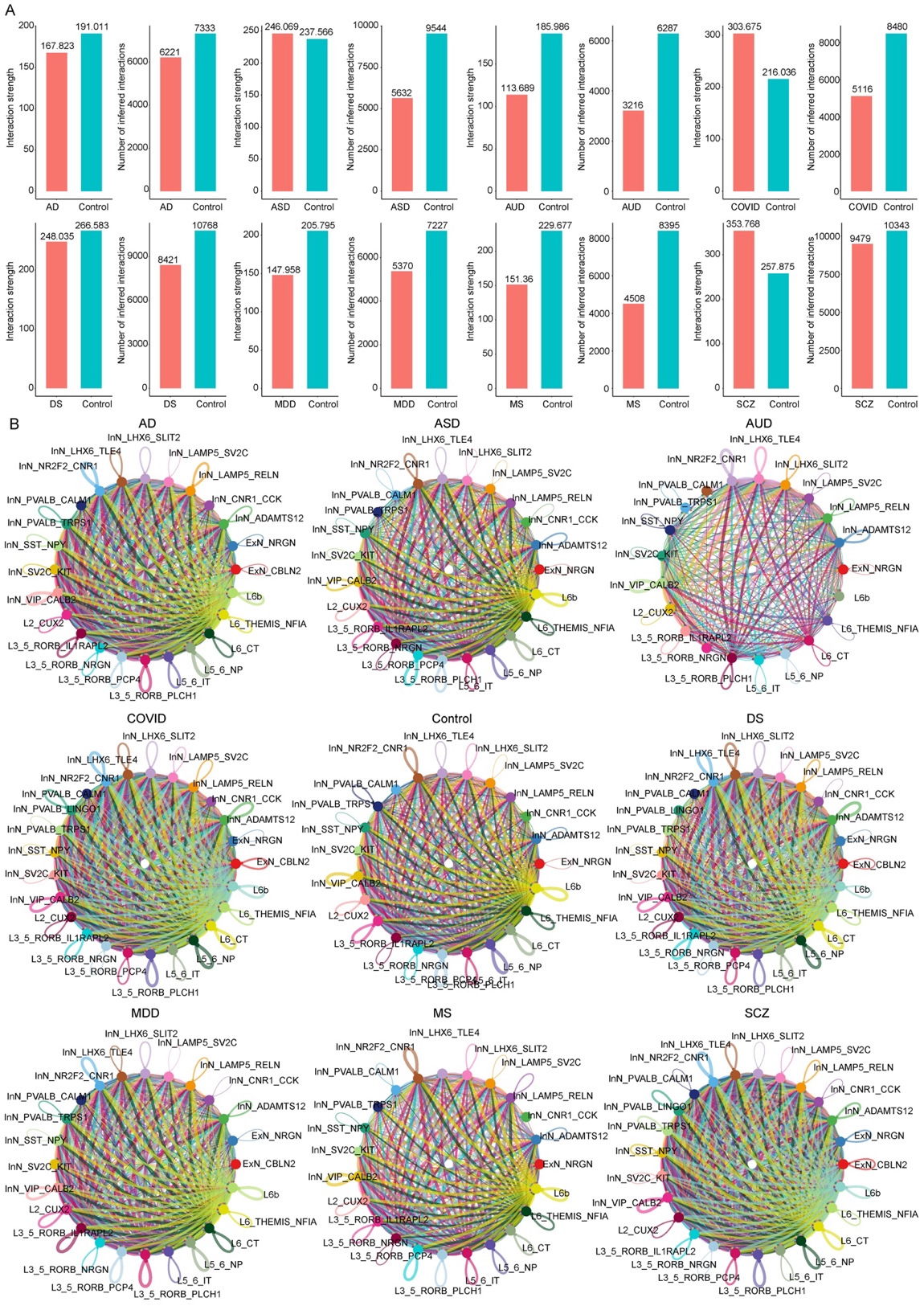
**

**Supplementary Figure.5. Comparison of interactions and neuronal subtype networks across diseases.** (A) Bar plots showing the strength and total number of interactions between ExN and InN in disease (AD, ASD, AUD, COVID, DS, MDD, MS, SCZ) versus control samples. (B) Circular network diagrams illustrating the neuronal subtype-specific interaction networks across diseases (AD, ASD, AUD, COVID, DS, MDD, MS, SCZ) and controls. Each node represents a neuronal subtype, and the edges represent interactions between subtypes. Line thickness indicates interaction strength. Nodes are color-coded by cell subtype, and interaction patterns reveal disease-specific alterations in neuronal networks.


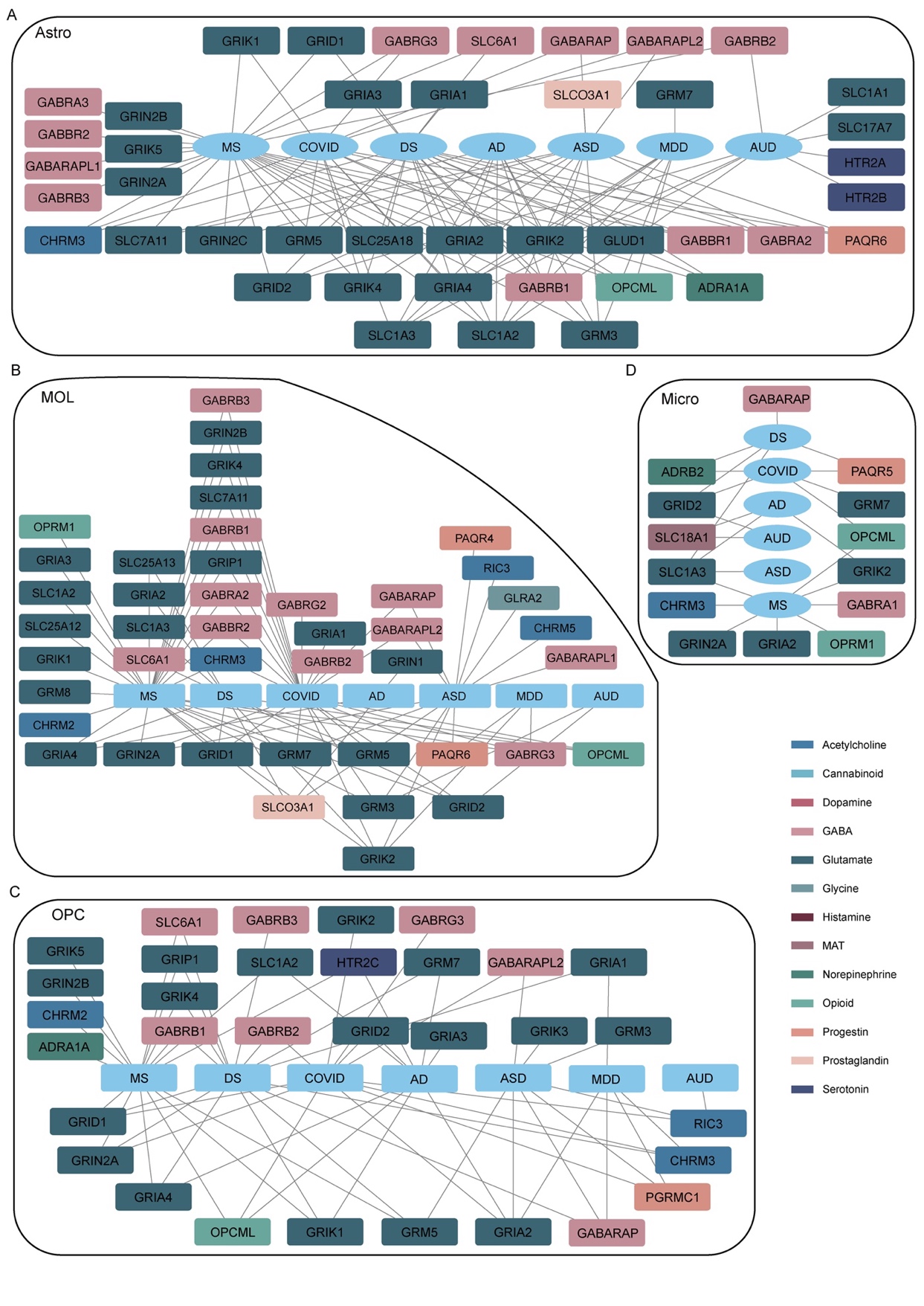


**Supplementary Figure. 6. NTS-related gene interaction networks in glial and OPCs across diseases.** Network visualization of NTS-related genes in Astro (A), MOL (B), OPC (C), and Micro (D) across diseases (MS, COVID, DS, AD, ASD, MDD, AUD). Nodes represent genes, color-coded by neurotransmitter system (e.g., GABA, glutamate, dopamine), while edges indicate interactions between genes. Blue ovals represent diseases, illustrating disease-specific gene interaction patterns.


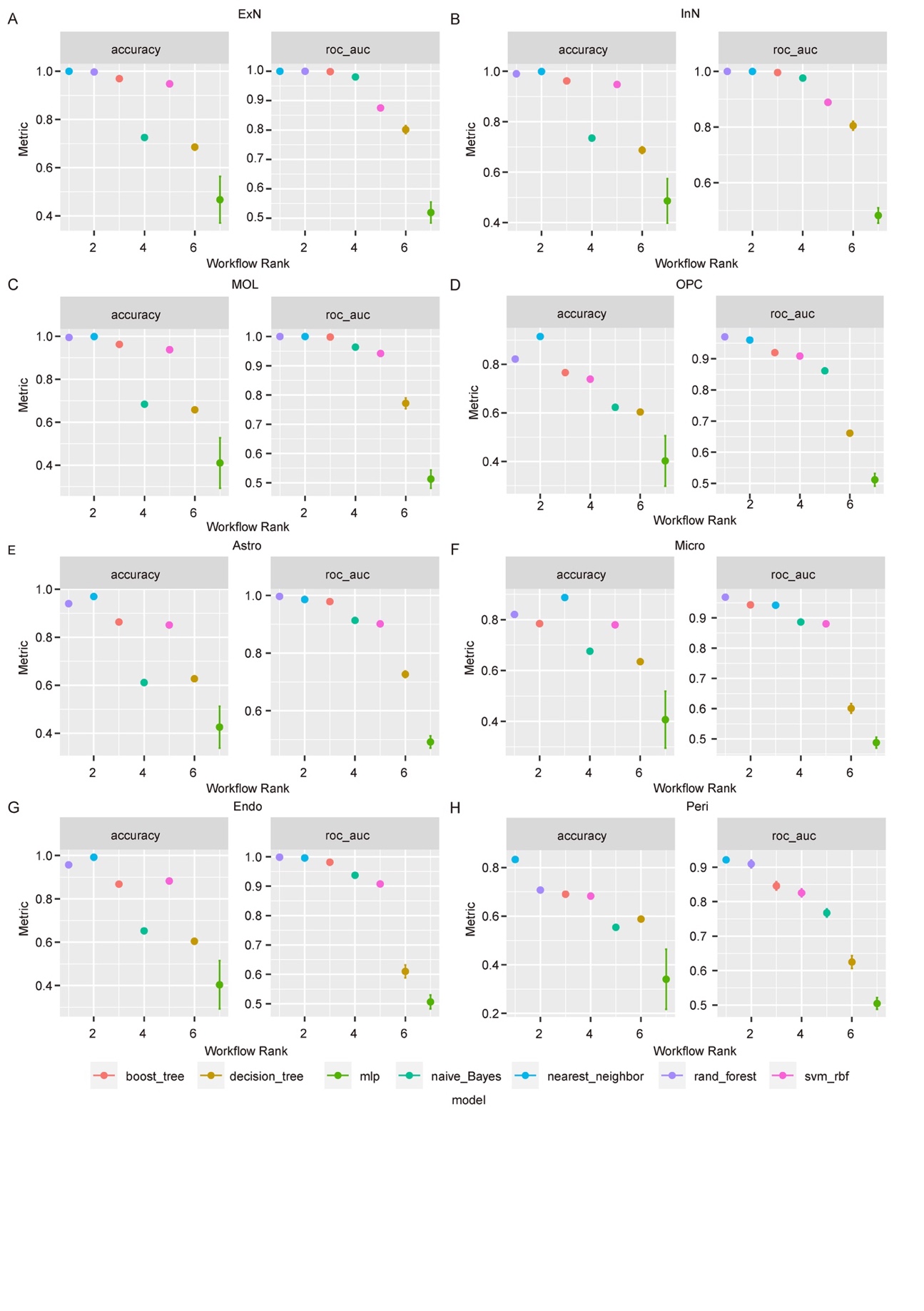


**Supplementary Figure. 7.** **Machine learning model performance in predicting cell disease states using NTS-related genes.** (A-H) Performance evaluation of different machine learning models for predicting disease states in various cell types based on neurotransmitter system (NTS)-related gene expression. Cell types analyzed include (A) ExN, (B) InN, (C) MOL, (D) OPC, (E) astrocytes, (F) microglia, (G) Endo, and (H) Peri. Each panel shows the metrics for model performance: Accuracy (left): The proportion of correctly predicted samples. ROC-AUC (right): The area under the receiver operating characteristic curve, indicating model discrimination ability. Machine learning models tested include boost_tree, decision_tree, mlp, naive_Bayes, nearest_neighbor, rand_forest, and svm_rbf. Points represent mean performance, and error bars indicate standard deviations.


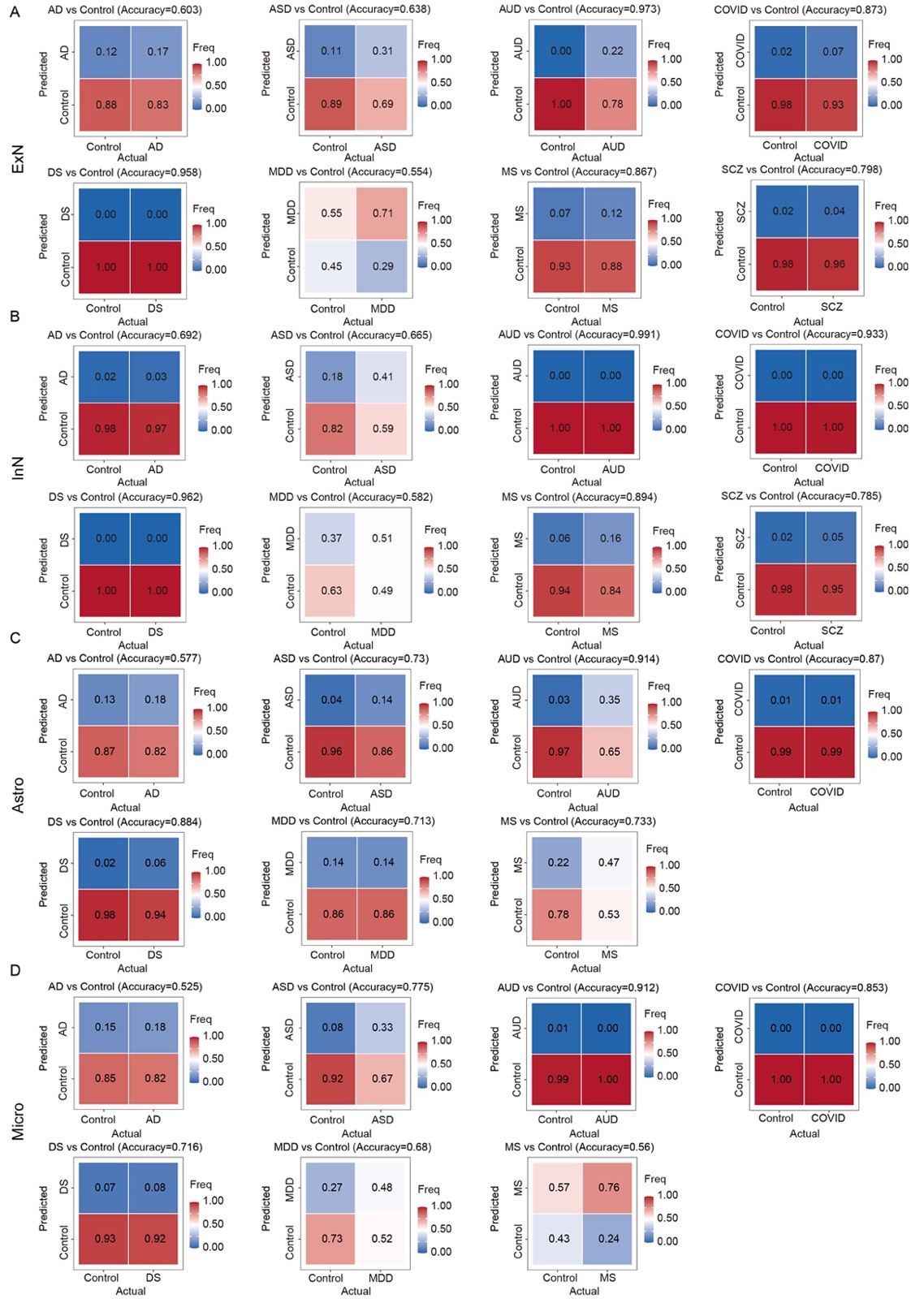


**Supplementary Figure. 8. Normalized confusion matrices for disease-versus-age-matched control classification in ExN, InN, Astro, and Micro.** Confusion matrices are shown for binary disease-versus-control classification in ExN (A), InN (B), Astro (C), and Micro (D) across the indicated disease groups. Models were trained using SampleID-aware partitioning, disease-specific age-matched controls, and neurotransmitter-system features after excluding Age from the predictive variables. Values in each cell represent the column-normalized frequency (0–1), and the corresponding test-set accuracy is shown above each matrix. These results are presented as a more stringent re-evaluation of classification performance after controlling for donor-level leakage and age-related confounding.


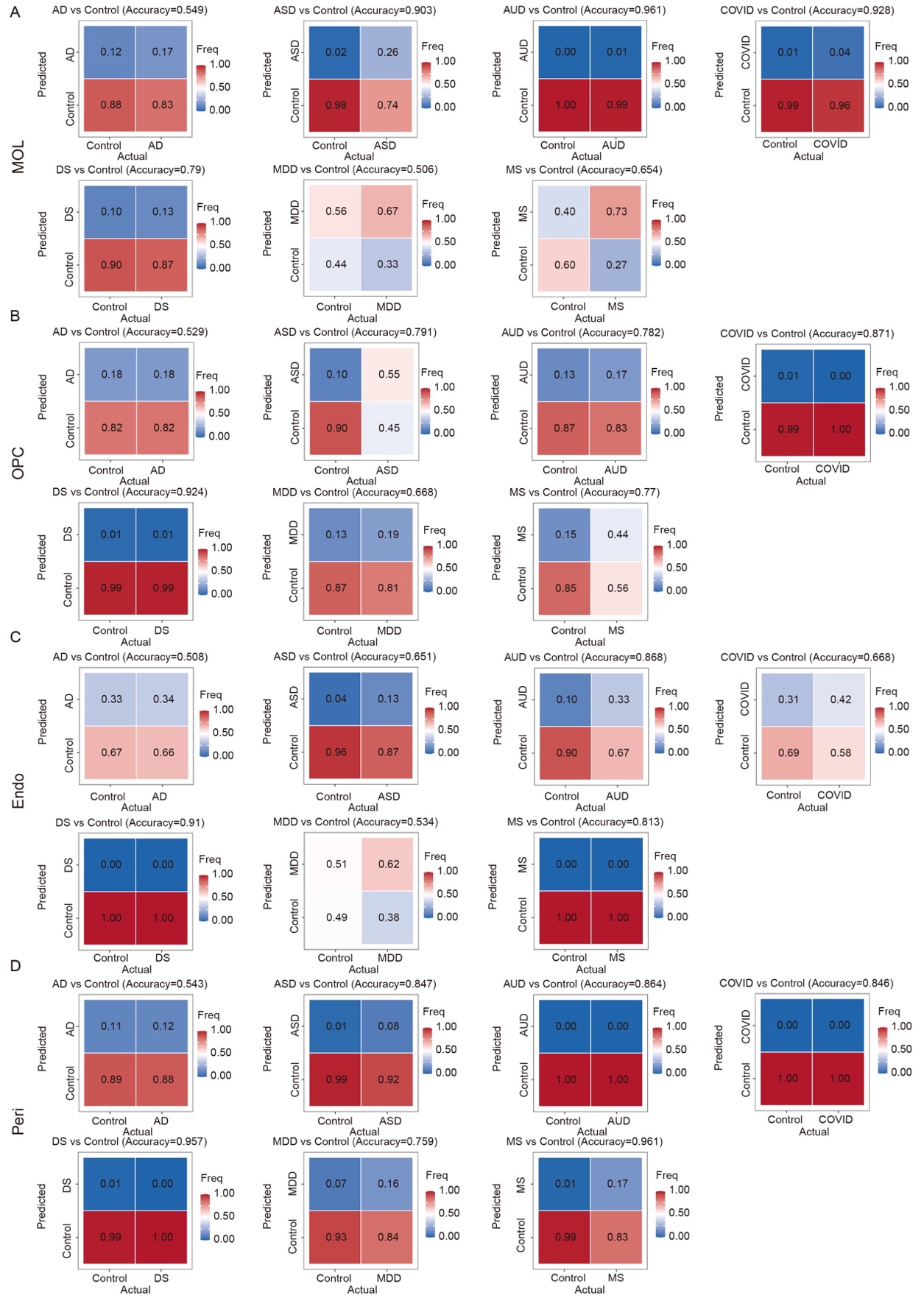


**Supplementary Figure. 9. Normalized confusion matrices for disease-versus-age-matched control classification in MOL, OPC, Endo, and Peri.** Confusion matrices are shown for binary disease-versus-control classification in MOL (A), OPC (B), Endo (C), and Peri (D) across the indicated disease groups. Models were trained using SampleID-aware partitioning, disease-specific age-matched controls, and neurotransmitter-system features after excluding Age from the predictive variables. Values in each cell represent the column-normalized frequency (0–1), and the corresponding test-set accuracy is shown above each matrix. These results are presented as a more stringent re-evaluation of classification performance after controlling for donor-level leakage and age-related confounding.


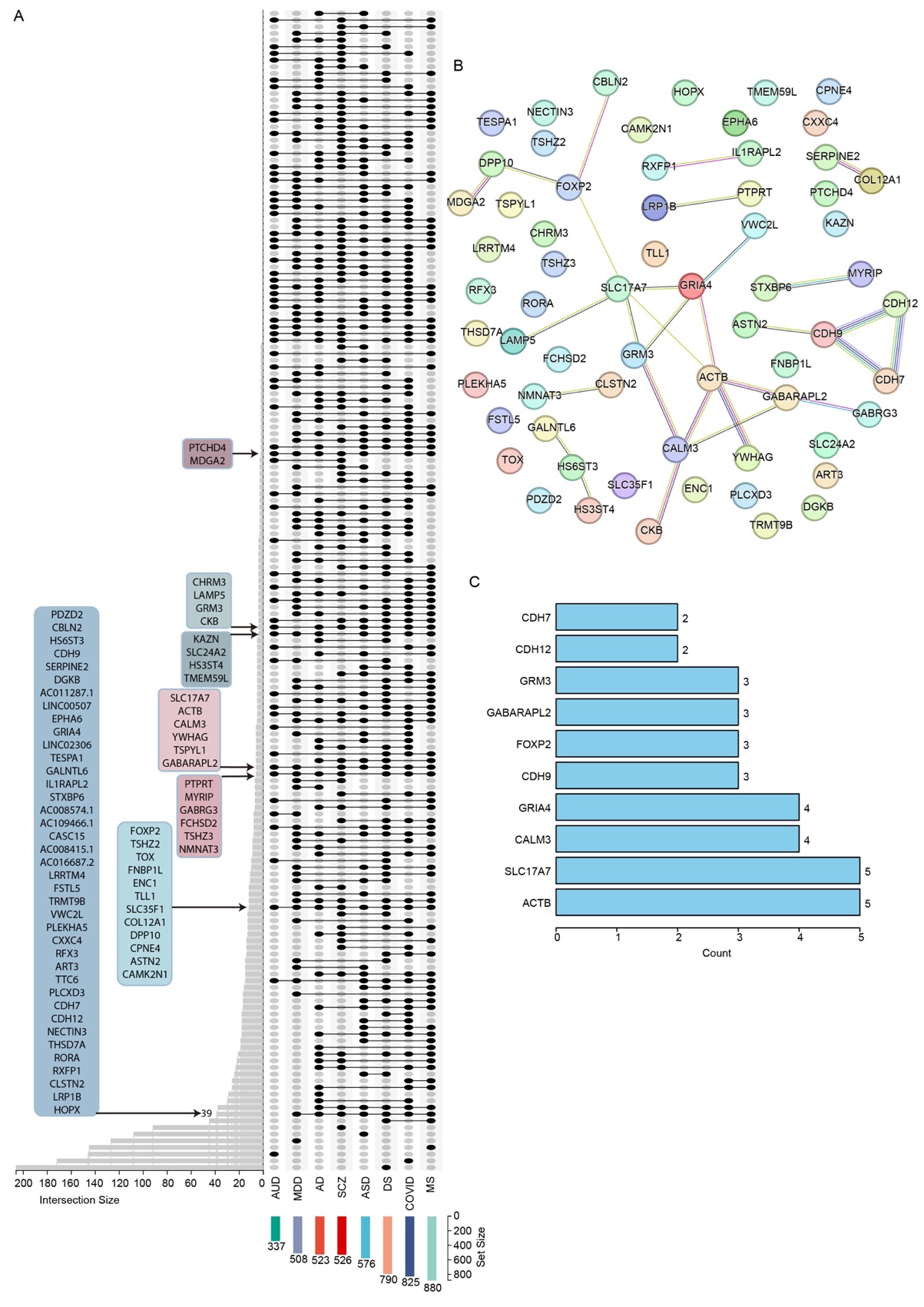


**Supplementary Figure. 10. Cross-disease analysis of NTS module-related genes.** (A) Upset plot of shared and unique NTS module-related genes across diseases. The horizontal bars represent the total number of genes identified in each disease (AD, ASD, SCZ, AUD, DS, MDD, MS, COVID). The vertical bars depict the size of gene intersections among different diseases, highlighting shared and unique gene sets. Selected key genes associated with multiple diseases are labeled. (B) PPI network of NTS-related genes altered in at least seven diseases. Nodes represent genes, and edges represent known protein-protein interactions. Highly connected genes in the network, such as GRIA4, ACTB, CALM3, and SLC17A7, are prominently featured, indicating their central roles in the network and potential relevance across diseases. (C) Bar plot of the top 10 highly connected genes in the PPI network. Genes are ranked based on their number of connections within the network. ACTB, SLC17A7, GRIA4, and CALM3 show the highest degree of connectivity, suggesting their significant involvement in the shared molecular mechanisms of multiple diseases.


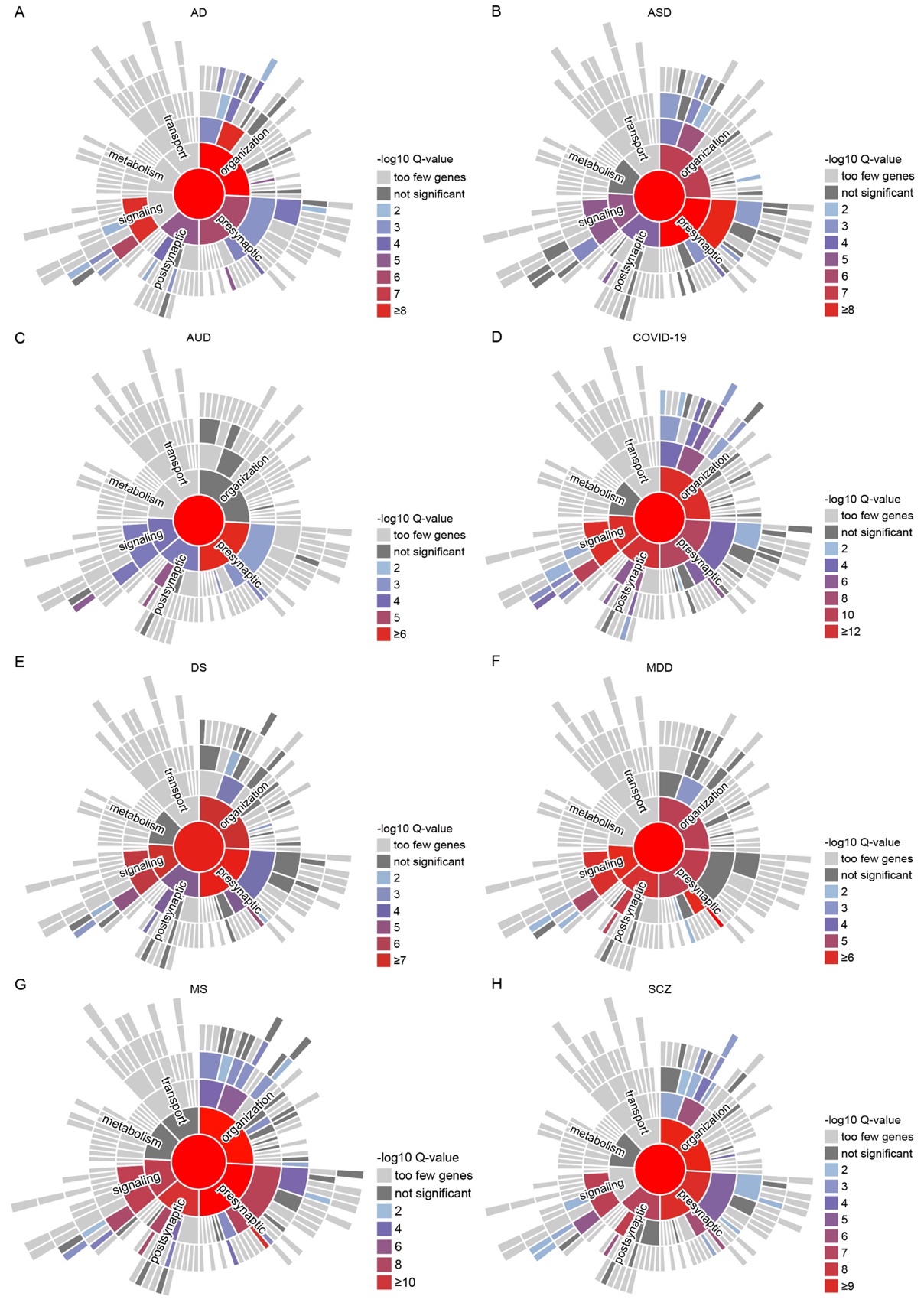


**Supplementary Figure. 11.** **SynGO analysis of NTS module-related genes in biological processes (BP) across diseases.** (A-H) Sunburst plots showing SynGO annotations of NTS module-related genes in synapse-related BP for each disease. Color intensity indicates the significance of enrichment (-log10 Q-value), with darker red representing higher significance. Gray segments indicate insufficient gene counts or non-significant enrichment.

**
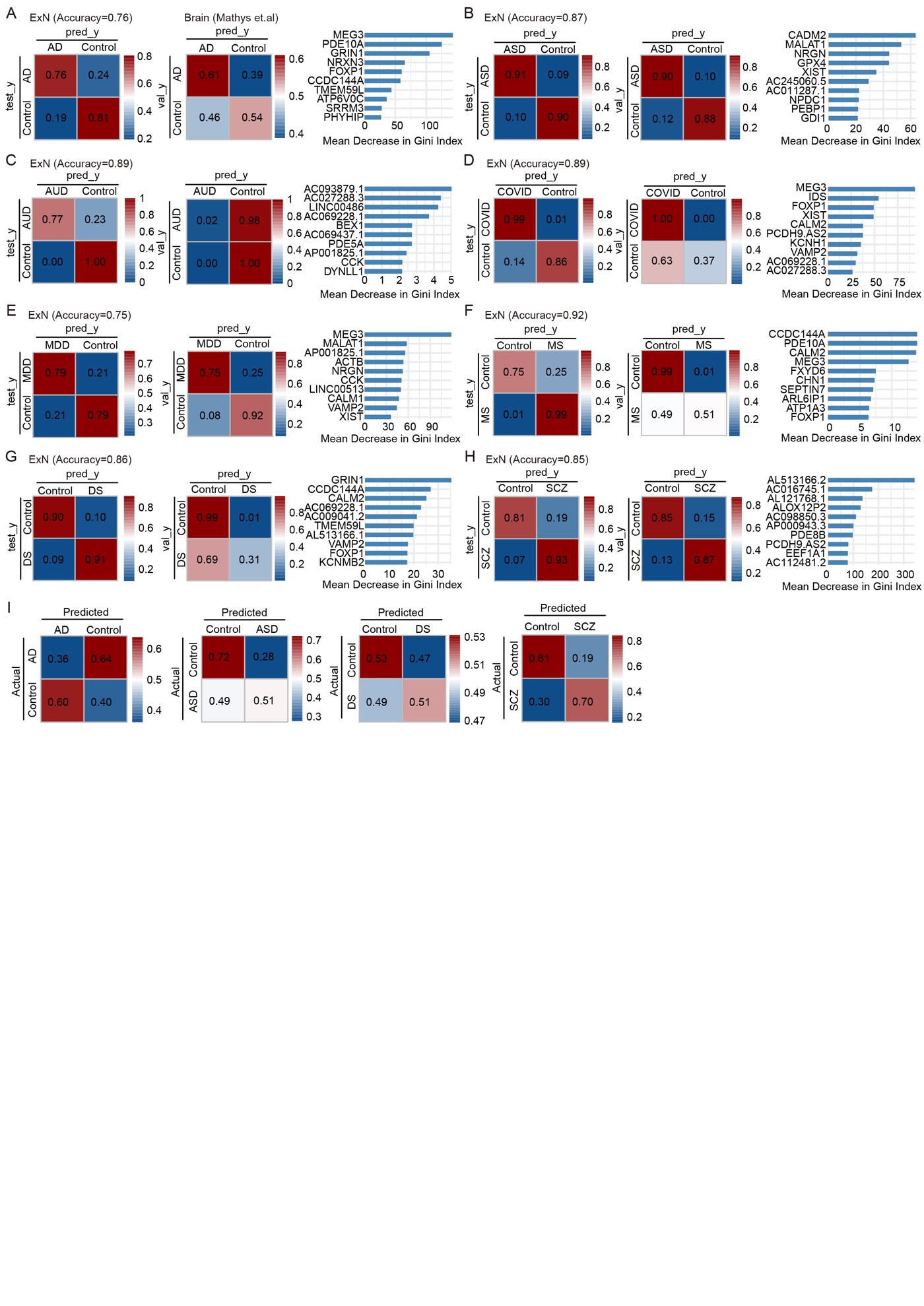
**

**Supplementary Fig. 12. Prediction of disease states using NTS module-related genes in ExN.** (A-H) Confusion matrices and feature importance for predicting control and disease states based on NTS module-related gene expression in ExN. Left panels: Confusion matrices showing the prediction performance, with color intensity indicating the proportion of correctly or incorrectly classified samples. The overall accuracy for each prediction is noted above each matrix. Right panels: Bar plots ranking the top 10 NTS-related genes contributing to the predictions, based on the mean decrease in Gini index. (I) Confusion matrices for external validation of module-gene-based classifiers in independent cerebral organoid datasets for AD, ASD, DS, and SCZ. These results provide supportive evidence that disease-associated module signals are partially recapitulated in organoid models.

**Supplementary Tables**

Supplementary Table 1

Sample database source and basic information.

Supplementary Table 2

The number of cell for cell type from different diseases.

Supplementary Table 3

NTS-related genes.

Supplementary Table 4

NTS related differentially expressed genes in different disease

Supplementary Table 5

SampleID-aware disease-versus-age-matched control classification performance across major cell types.

Supplementary Table 6

NTS module-related genes for ExN in different diseases.
